# Inhaled Macrophage Apoptotic Bodies-Engineered Microparticle Enabling Construction of Pro-Regenerative Microenvironment to Fight Hypoxic Lung Injury in Mice

**DOI:** 10.1021/acsnano.4c03421

**Published:** 2024-05-10

**Authors:** Chang Liu, Xingping Quan, Xidong Tian, Yonghua Zhao, Hai-Feng Li, Judith Choi Wo Mak, Zhenping Wang, Shirui Mao, Ying Zheng

**Affiliations:** † State Key Laboratory of Quality Research in Chinese Medicine, Institute of Chinese Medical Sciences, University of Macau, Macau999078, China; ‡ Department of Pharmaceutical Sciences, Faculty of Health Sciences, University of Macau, Macau999078, China; § Joint Key Laboratory of the Ministry of Education, Institute of Applied Physics and Materials Engineering, University of Macau, Macau999078, China; ∥ Department of Pharmacology and Pharmacy, LKS Faculty of Medicine, The University of Hong Kong, 999077, Hong Kong, China; ⊥ Department of Dermatology, School of Medicine, University of California, San Diego, California92093, United States; # School of Pharmacy, Shenyang Pharmaceutical University, Shenyang110016, China; ∇ Joint International Research Laboratory of Intelligent Drug Delivery Systems, Ministry of Education, Shenyang 110016, China

**Keywords:** hypoxic acute lung injury, pulmonary drug delivery, membrane camouflaged microparticle, alveolar epithelial cells repair, hyper-inflammation inhibition

## Abstract

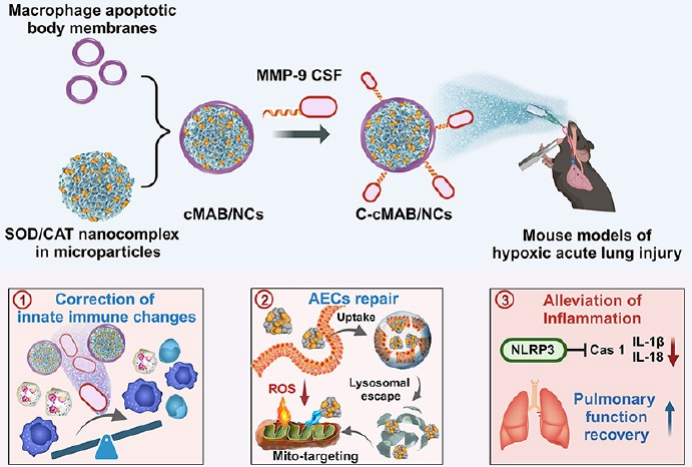

Oxygen therapy cannot rescue local lung hypoxia in patients with severe respiratory failure. Here, an inhalable platform is reported for overcoming the aberrant hypoxia-induced immune changes and alveolar damage using camouflaged poly(lactic-*co*-glycolic) acid (PLGA) microparticles with macrophage apoptotic body membrane (cMAB). cMABs are preloaded with mitochondria-targeting superoxide dismutase/catalase nanocomplexes (NCs) and modified with pathology-responsive macrophage growth factor colony-stimulating factor (CSF) chains, which form a core–shell platform called C-cMAB/NC with efficient deposition in deeper alveoli and high affinity to alveolar epithelial cells (AECs) after CSF chains are cleaved by matrix metalloproteinase 9. Therefore, NCs can be effectively transported into mitochondria to inhibit inflammasome-mediated AECs damage in mouse models of hypoxic acute lung injury. Additionally, the at-site CSF release is sufficient to rescue circulating monocytes and macrophages and alter their phenotypes, maximizing synergetic effects of NCs on creating a pro-regenerative microenvironment that enables resolution of lung injury and inflammation. This inhalable platform may have applications to numerous inflammatory lung diseases.

The acute respiratory distress syndrome (ARDS) is a life-threatening condition characterized by noncardiogenic pulmonary edema and hypoxaemia, with high levels of morbidity and mortality.^1^ Despite the standard therapy with lung-protective ventilation, ARDS patients had clinical evidence of persistent hypoxemia during the first 48 h of ventilation.^2^ Unfortunately, effective therapies to rescue local lung hypoxia and inhibit ensuing inflammatory injury are lacking. Levels of hypoxia are inevitably exacerbated in lung injury by an imbalance between oxygen supplement and consumption; supplement is disrupted due to loss of alveolar basement membrane integrity, while consumption is promoted due to metabolic disorders triggered by the lung tissue resident cells and infiltrating immune cells.^3,4^ To maximize efficacy, we postulate that therapies repairing alveolar integrity and correcting the aberrant hypoxia-induced immune changes are promising interventions.

Impaired lung epithelial function has been implicated in the complex pathogenesis of ARDS,^5^ and recent studies suggest that mitochondrial dysfunction is predominantly responsible for cell death,^6^ which ultimately contribute to epithelial barrier disruption. More specifically, excess accumulation of mitochondrial reactive oxygen species (mtROS) results in activation of the NOD-like receptor thermal protein domain associated protein 3 (NLRP3)/caspase-1 mediated pathway, continuing to aggravate the lung injury.^7,8^ Hence, targeting mitochondrial dysfunction in alveolar epithelial cells (AECs) may be an effective strategy to combat alveolar damage. Therapies like antioxidants have been leveraged to scavenge ROS, which are helpful for protecting cells against oxidative damage.^9^ Nevertheless, because of their rapid clearance and insufficient cellular internalization after administration,^10^ antioxidants demonstrate relatively inefficient mtROS-scavenging ability and inconsistent efficacy for restoration of cell function.

In addition to alveolar damage, patients with ARDS developed profoundly monocytopenia, with a failure to expand monocyte-derived macrophages and persistent inflammation.^11,12^ Recently, research efforts have confirmed the effect of monocyte and macrophage growth factor colony-stimulating factor 1 (CSF-1) on correcting these hypoxia-mediated immune changes, indicating the therapeutic benefit of CSF-1 in ARDS.^13^ Unfortunately, these growth factors, as biologic therapeutics, are conventionally administered systemically via intravenous injection, which limits their accumulation in targeted lung tissues, leading to decreased therapeutic efficacy.^14^ Furthermore, one must also be aware of the potential risk of CSF-1 treatment for systemic side effects.^15^

Herein, we developed an inhalable platform that could sustainably inhibit inflammasome-mediated AEC damage while also improving CSF delivery for innate immune cell reprogramming in lung injury (Scheme 1). Our platform (C-cMAB/NC) was designed as follows (Scheme 1): (i) superoxide dismutase (SOD)/catalase (CAT) nanocomplexes (NCs) for the intracellular mitochondrial-targeted delivery of antioxidants, (ii) a matrix metalloproteinase 9 (MMP-9)-cleavable CSF shell to perform accurate CSF release in deeper alveoli for enhancing immunotherapy, and (iii) a chimeric macrophage apoptotic body (cMAB) core fabricated by a natural macrophage apoptotic body membrane (MABM) and a modular poly lactic-*co*-glycolic acid (PLGA) microparticle (MP). To maximize synergistic efficacy following inhalation, we sequentially assembled the platform (C-cMAB/NC) by using a cMAB core preloaded with SOD/CAT NCs and modified with a cleavable CSF shell. Research efforts have advanced the understanding of the role for macrophage-derived ABs in intercellular communication with AECs in response to LPS stimuli.^16,17^ Therefore, by using the natural membrane of ABs derived from macrophages,^18^ the cMABs that we fabricated not only specifically delivering SOD/CAT NCs to AECs, but they were also desirable for conjugating with CSF shells via click reaction without affecting the CSF biological function. Inhalation of C-cMAB/NCs promoted the pulmonary codelivery of multiple therapeutics into deeper lung alveoli, where the outer CSF shell cleavage by MMP-9 and the cMAB/NC core exposure specifically delivered NCs into mitochondria within AECs. Note that we selected the combination of SOD and CAT, known as the front line of antioxidants, to scavenge mtROS enabled by the tannic acid (TA)-mediated self-assembly of NCs.^19^ In principle, the cascade catalysis toward cytotoxic ROS by the combination of SOD and CAT not only displays incomparable ROS scavenging efficiency but also contributes to minimizing other reactive oxygen or nitrogen species production that many small-molecule antioxidants commonly encounter.^20^ Therefore, pulmonary delivery of nano-SOD/CAT and CSF to their specific action sites directly modulated the lung hyper-inflammatory microenvironment into a pro-regenerative state, contributing to a reshaping of innate immune cells and inhibition of inflammasome-mediated AEC damage. Note that these effects had a sustained impact on the resolution of lung injury and inflammation in mouse models of hypoxic acute lung injury (HALI).

**Scheme 1:**
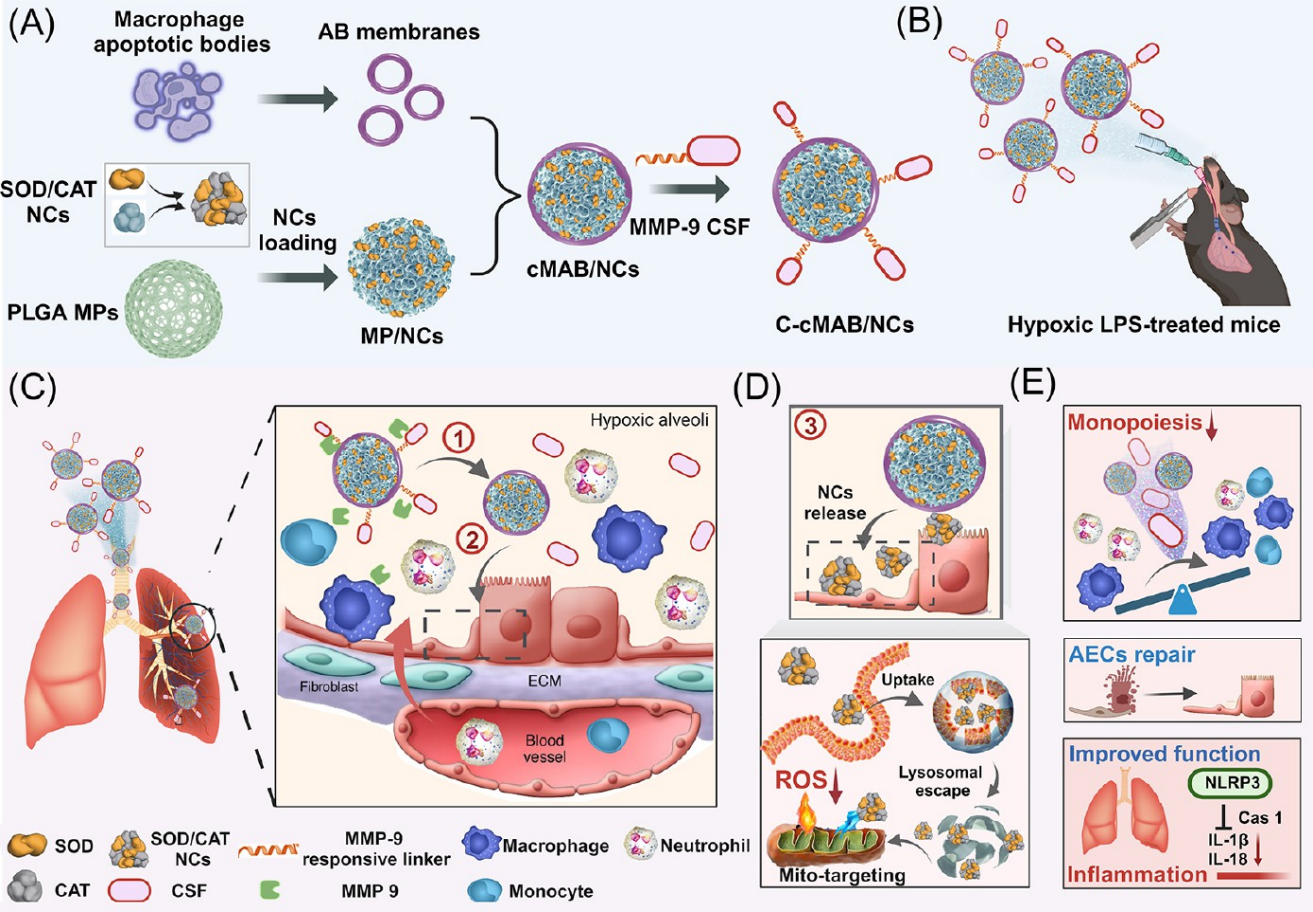
Schematic Illustration of an Inhalable Cascade-targeting, Multiple Therapeutics–Loaded, Core–Shell Platform (C-cMAB/NC) for HALI Treatment (Created with BioRender.com and Depositphotos.com) (A) Illustration of C-cMAB/NCs construction. (B) Inhalation of C-cMAB/NCs in mouse models of HALI. (C) C-cMAB/NCs displayed efficient deposition in deeper alveoli and high affinity to AECs after CSF shell cleavage by MMP-9. (D) Partial release and cellular internalization of NCs from the cMAB/NCs. After lysosomal escape, NCs targeted mitochondria and efficiently scavenged mtROS. (E) Inhalation of C-cMAB/NCs rescued the monocytopenia and inhibited the inflammasome-mediated AECs damage, pros of HALI.

## Results and Discussion

### Preparation and Characterization of C-cMAB/NCs

**Figure 1:**
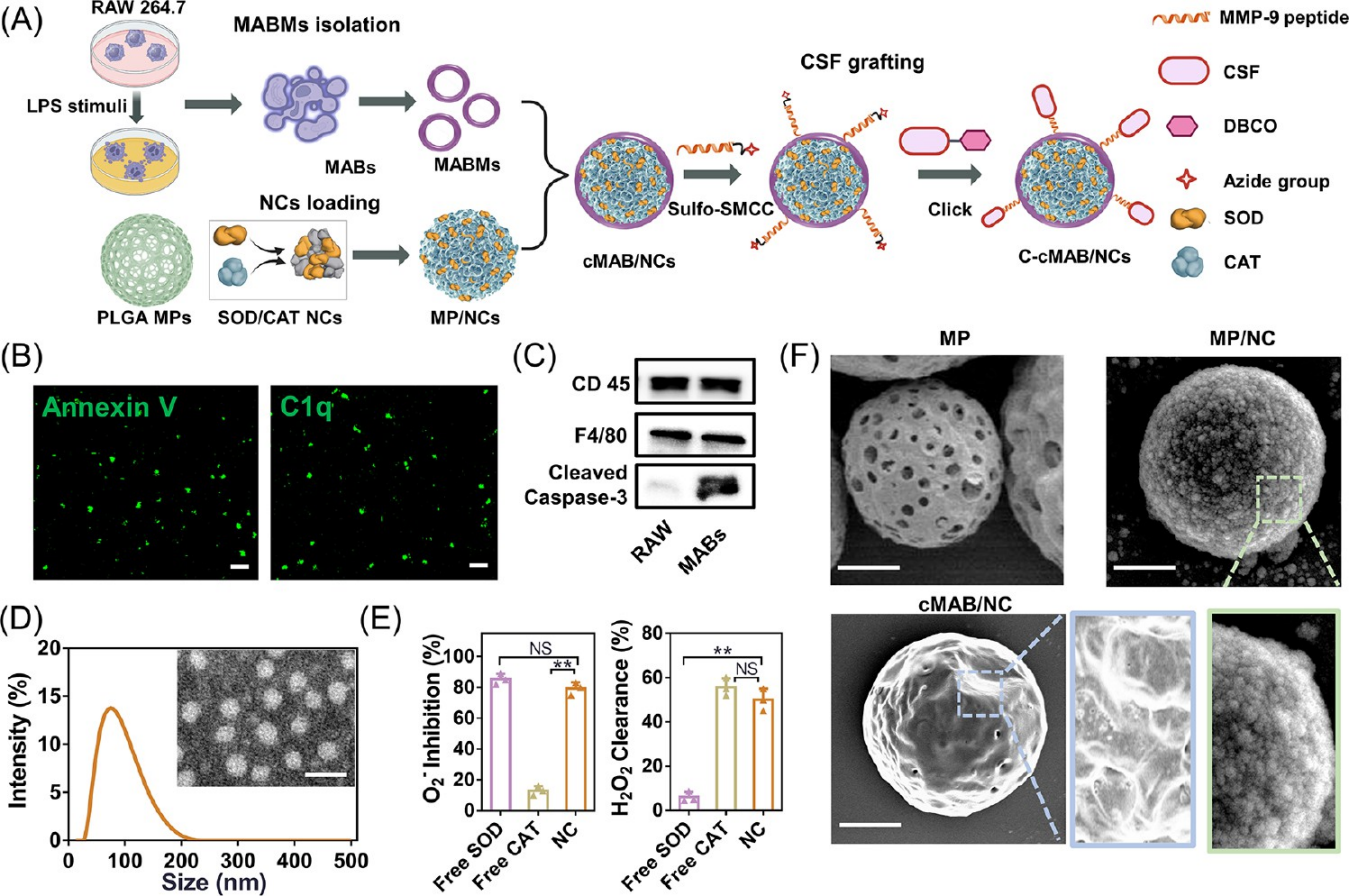
Construction and characterization of C-cMABs (created with BioRender.com). (A) Fabrication of the cascade-targeting, multiple therapeutics–loaded, core–shell platform (C-cMAB/NC). (B) Annexin V and C1q staining of MABs. Scale bars: 20 μm. (C) Representative protein profiles of RAW 264.7 cells and MABs. (D) Size distribution and representative TEM image of NCs. Scale bars, 100 nm. (E) Scavenging of superoxide anion and hydrogen peroxide by NCs (*n* = 3). (F) Representative SEM images of PLGA MPs, MP/NCs and cMAB/NCs. Scale bars, 5 μm. (Inset) Zoomed-in area of the previous image. **p < 0.01, n.s., not significant, *p* > 0.05.

**Figure 2:**
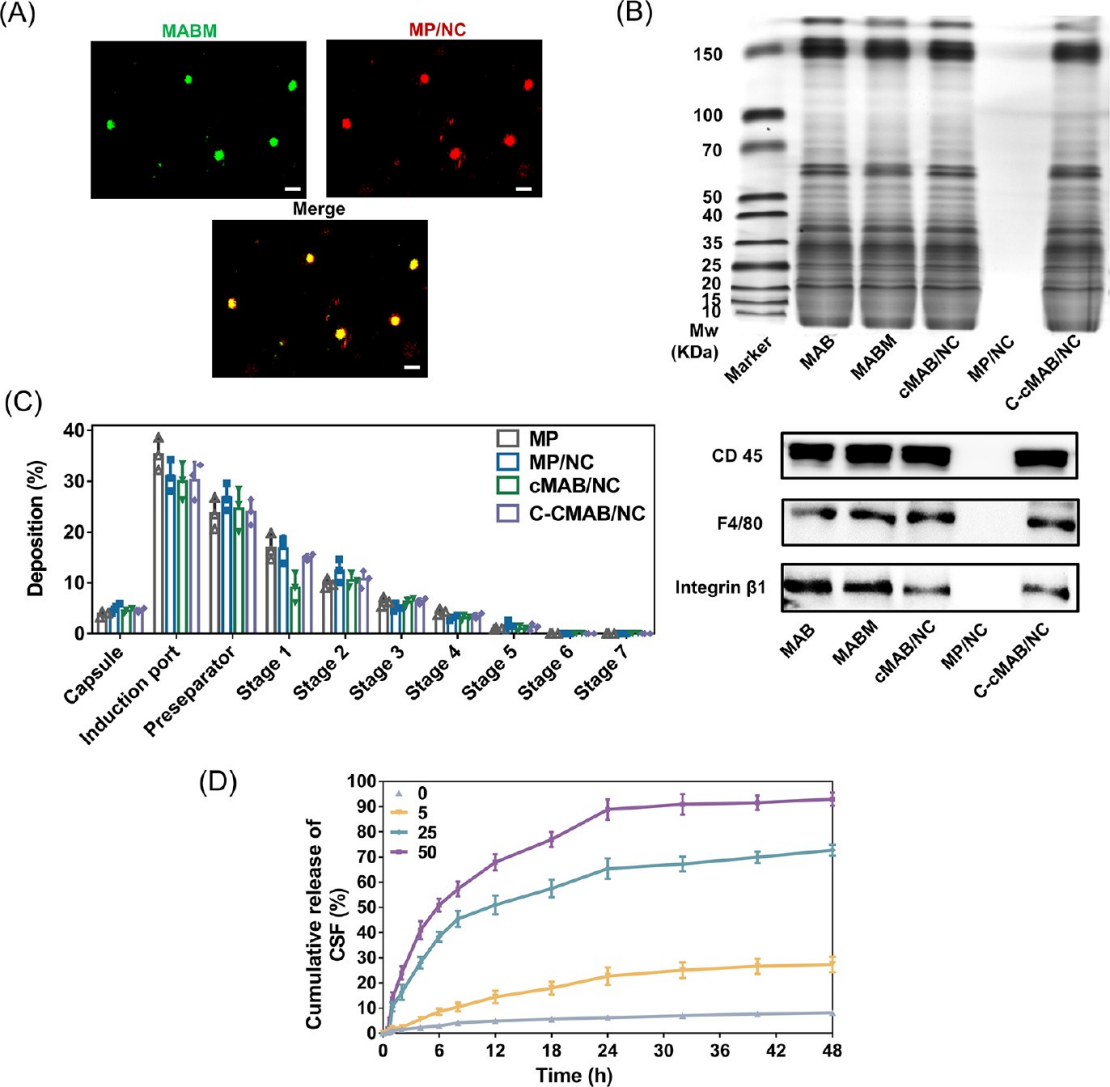
Characterization of C-cMABs. (A) Confocal images of the cMAB/NCs. MABMs were labeled with FITC (green), and MP/NCs were labeled with Cy5.5 (red). Scale bars, 20 μm. (B) Protein profiles visualization of MABs, MABMs, cMAB/NCs, MP/NCs, and C-cMAB/NCs by Coomassie Blue staining and Western blotting analysis. (C) *In vitro* aerodynamic particle size distribution of microparticles measured by NGI. (D) Cumulative CSF release from C-cMAB/NCs in medium with various concentrations of MMP-9 (0, 5, 25, 50 nM) (*n* = 3).

The cascade-targeting, multiple therapeutics–loaded, core–shell platform (C-cMAB/NC) consisting of an MABM camouflaged PLGA core (cMAB) loaded with NCs and a cleavable CSF shell was fabricated by third steps, as shown in Figure 1A. First, the successful induction of apoptosis in RAW 264.7 cells was confirmed by the expression of Annexin V and C1q (Figure 1B; Figure S1A,B). These results were also supported by analysis of apoptosis-related proteins such as cleaved caspase3 for MABs (Figure 1C). We therefore isolated intact MABM vesicles from MABs by a combination of hypotonic lysis and sonication. The MABM vesicles displayed a spherical morphology with a size of approximately 1 μm (Figure S1C), which was consistent with the DLS results (Figure S1D). Next, the inner core of our platform was fabricated by loading nano-SOD/CAT into PLGA MPs that were subsequently camouflaged with the MABM vesicles (Table S1). Considering that the combination of SOD and CAT has hardly any access to mtROS due to its insufficient cellular internalization, we prepared mitochondria-targeting NCs by inducting the self-assembly of SOD and CAT in water with the aid of TA. The resulting NCs displayed a spherical morphology with size of 50 nm and zeta potential of −9 mV, as assessed by TEM and DLS, respectively (Figure 1D). The loading capacities (DLCs) were calculated to be 45.2 ± 2.1% for SOD and 65.3 ± 2.2% for CAT. The *in vitro* ROS scavenging results directly revealed the satisfying enzymatic activity of NCs, with 92.1% of the SOD enzymatic activity remaining (Figure 1E). Likewise, about 94.2% of the CAT enzymatic activity could be kept in NCs relative to free CAT. To avoid unwanted release before internalization by target cells, NCs were encapsulated into biomimetic MPs with MABM modification. The resulting cMAB/NCs performed a spherical morphology with an obvious membrane coating on the MP (Figure 1F) and no visible surface pore areas (Figure S1E). Representative CLSM images showed a good colocalization of MABM (green) and MP/NC (red) fluorescent signals on cMAB/NCs (Figure 2A), confirming the successful MABM coating. After our calculation, the membrane coating efficiency of cMAB/NCs reached 93.3% (Figure S1F), again demonstrating an effective shielding of MP/NCs by MABM coating. Coomassie blue staining and Western blotting analysis (Figure 2B) confirmed the good retention of MABM protein profiles on cMAB/NCs. Finally, an MMP-9 responsive CSF shell was grafted onto the surface of cMAB/NCs via click reaction to achieve accurate CSF release in damaged alveoli without influencing its activity. The corresponding C-cMAB/NCs showed an effective CSF grafting with an encapsulation efficiency (EE) of 42.6%. Additionally, the activity of SOD used in the formulation was 5801 ± 10 U/mg and that of CAT was 4940 ± 21 U/mg. The EE of SOD was 79.3 ± 4.6%, whereas that of CAT was 84.9 ± 4.2% (Table S1). Based on the assay kit, each mg of C-cMAB/NC contained 507.9 ± 4.6 units or ∼88 μg of SOD and 581.6 ± 4.5 units or ∼118 μg of CAT. Furthermore, CSF leakage from the C-cMAB/NCs was evaluated by ELISA, where the EE of CSF had no significant change at day 7 of 4 °C storage (Figure S1G). Coomassie blue staining and Western blotting analysis (Figure 2B) revealed the negative effect of CSF grafting on the protein profiles of the cMAB/NCs.

It has been well demonstrated that particle size, referred to as an aerodynamic diameter, being the most important characteristic for determining particle deposition site in the respiratory system.^21^ Hence, by design, we produced C-cMAB/NCs with an aerodynamic diameter of ∼3.9 μm for fulfilling effective deposition of loaded therapeutics in the deeper alveoli (Table S1 and Figure 2C). We next assessed the *in vitro* release profile of C-cMAB/NCs in a medium containing different concentrations of MMP-9 that mimics the alveoli hyper-inflammatory environment to determine the MMP-9 responsiveness of C-cMAB/NCs. As shown in Figure 2D, cumulative CSF release from C-cMAB/NCs could reach 89.2% in the presence of 50 nM MMP-9 after a 48 h incubation, which was in marked contrast to a slight release of CSF (9.2 ± 2.5%) in the MMP-9 free medium. These results indicated that C-cMAB/NCs had a high MMP-9 sensitivity, ensuring controlled CSF release in the injured alveoli, which is desirable for improving immunotherapy.

### Delivery of NCs into Mitochondria within AECs

**Figure 3:**
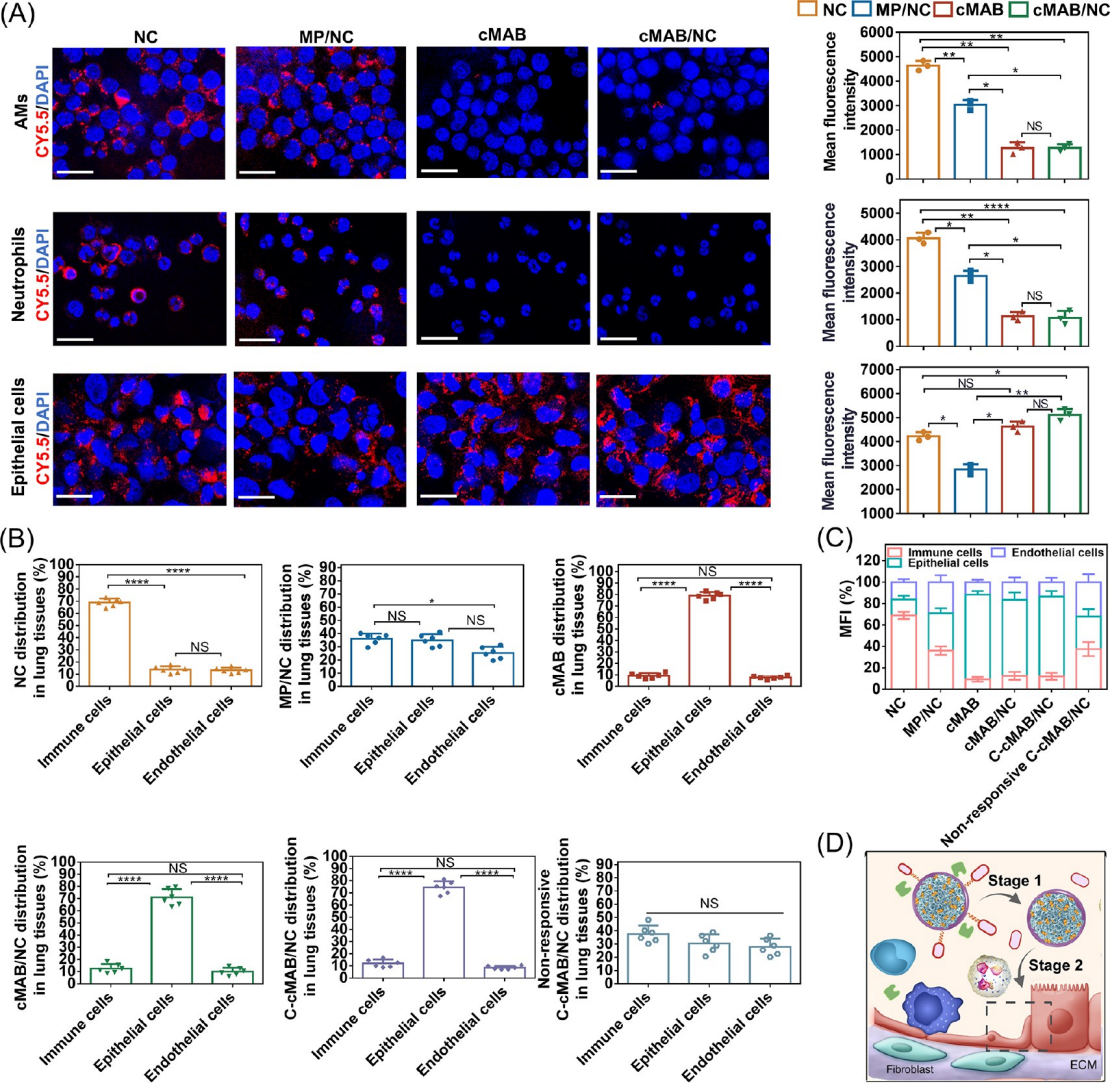
High affinity to AECs endowed by MABM coating (created with BioRender.com and Depositphotos.com). (A) Confocal images and colocalization analysis of primary AMs, neutrophils, and MLE-12 cells incubated with NCs, MP/NCs, cMABs, or cMAB/NCs for 2 h (nuclei stained by Hoechst (blue), Cy5.5-labeled formulations (red); *n* = 3). Scale bar, 20 μm. (B, C) Co-localization analysis of cells from the lung tissues treated with Cy5.5-labeled NCs, MP/NCs, cMABs, cMAB/NCs, C-cMAB/NCs, or MMP-9 nonresponsive C-cMAB/NCs (*n* = 6). (D) Schematic illustration of the selectively targeting property of C-cMAB/NCs in the lung of LPS-treated hypoxic mice. *p < 0.05, **p < 0.01, ****p < 0.0001, n.s., not significant, *p* > 0.05.

Evidence is emerging for key roles of LPS-induced, macrophage-derived ABs in intercellular communication with AECs.^22^ Considering the selective cellular targeting ability of MABs, we started to examine the *in vitro* interaction of cMAB/NCs before and after CSF grafting with MLE-12 cells (mimicking *in vivo* target cells) and typical lung phagocytes, including AMs and neutrophils. First, the F4/80 and CD11c double-positive mouse AMs as well as Gr1 and CD11b double-positive mouse neutrophils were identified by flow cytometry analysis and Diff-Quik Staining (Figure S2A), respectively. Confocal images showed that cMAB/NCs produced weak red fluorescence in AMs and neutrophils but an obviously enhanced red signal in MLE-12 cells compared to MP/NCs without membrane modification or bare NCs (Figure 3A). Quantitative analysis of cellular uptake based on flow cytometry again emphasized that the MABM coating strategy endowed MP/NCs with a high affinity to AECs. This may be due to the expression of integrin β1 on MABMs, which involved in AB/recipient epithelial cell contact.^23^ In the presence of MMP-9, treatment of the AMs, neutrophils, and MLE-12 cells with C-cMAB/NCs showed the same trends as the cMAB/NC group, whereas this effect was reversed in the cells treated with MMP-9 nonresponsive C-cMAB/NCs (Figure S2B). This was attributed to the cleavage of CSF shells by MMP-9, leading to the cMAB/NC core exposure for interaction with AECs. Note that the fluorescence signal of cMABs was also detected using Cy5.5-labeled MABM coating. The results showed that the Cy5.5 fluorescence signal mostly distributed around the cell nuclei over time, indicating that the NC release process out of the cMAB carrier could be visualized, where not the cMABs themselves but loaded NCs were internalized by the cells (Figure S4A). Afterward, we examined the majority cell subtype in the hypoxic lung that C-cMAB/NCs entered after intratracheal administration. Analysis of markers for AECs, endothelial cells, and immune cells in the mouse lungs indicated that approximately 74.5% of NCs were colocalized with AECs in the lung with C-cMAB/NC or C-cMAB/NC treatment, while much fewer NCs were delivered into AECs in the lung from the naked NCs or MMP-9 nonresponsive C-cMAB/NCs treated group (Figure 3B,C; Figure S3A,B). In agreement with *in vitro* uptake studies, these results clarified that inhalation of C-cMAB/NCs contributed to CSF shells cleavage and cMAB/NC core exposure within the inflamed alveoli, leading to efficiently and selectively delivering NCs into AECs (Figure 3D).

**Figure 4:**
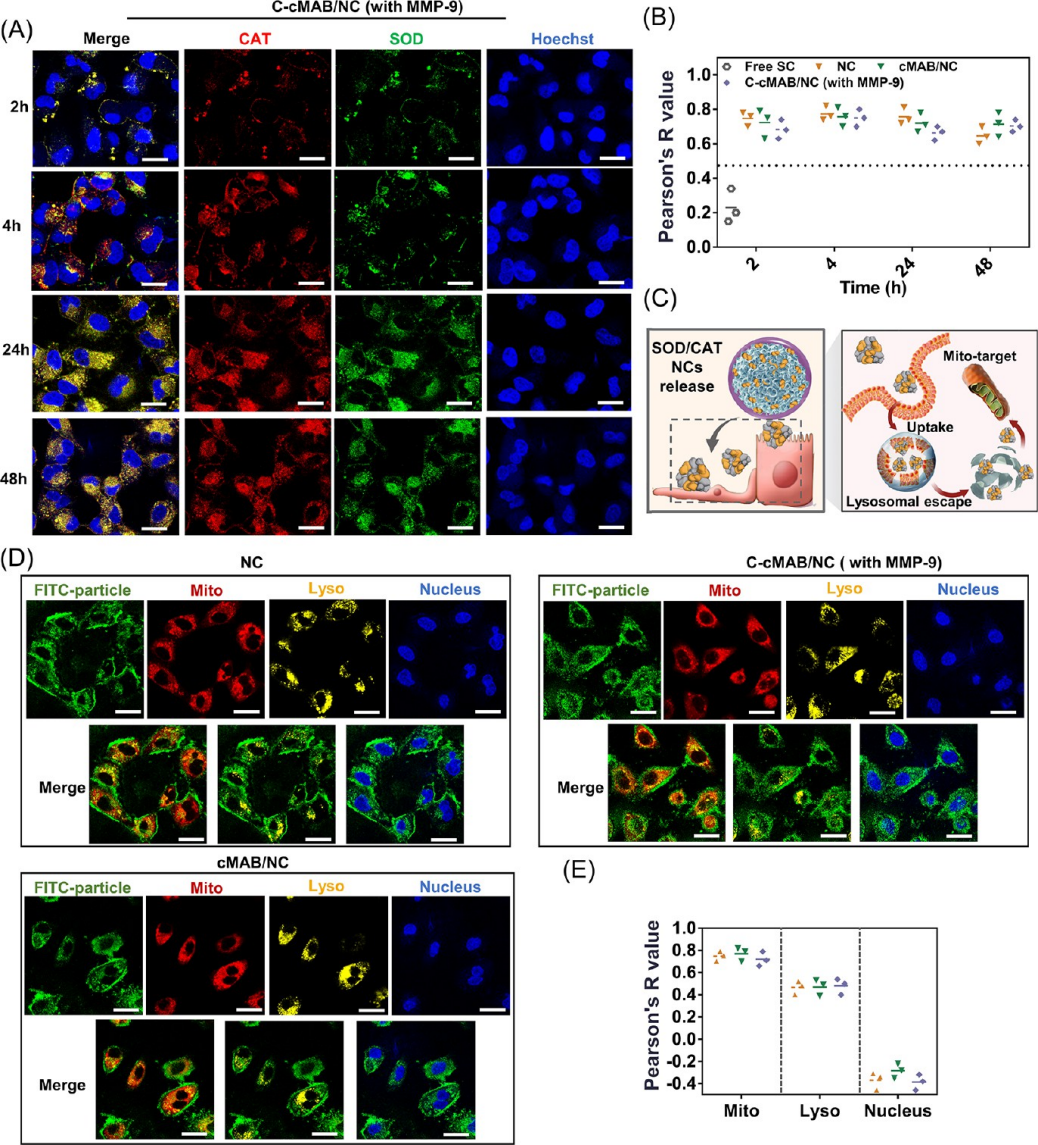
Cascade-targeting evaluation of C-cMAB/NCs (created with BioRender.com). (A) Confocal images of MLE-12 cells taken for evaluating NCs release after 2, 4, 24, and 48 h of incubation (nuclei stained by Hoechst (blue), Cy5.5-labeled CAT (red) and FITC-labeled SOD (green) used for colocalization). Scale bar, 20 μm. (B) Image-based colocalization analysis of SOD and CAT at indicated time points. R indicates the Pearson’s correlation coefficient of red fluorescence with green fluorescence. (C) Schematic illustration of the cell-permeable mitochondria-targeting property of C-cMAB/NCs in the presence of MMP-9. (D) Subcellular localization of the FITC-labeled NCs in the first 24 h after incubation (nuclei stained by Hoechst (blue), mitochondria stained by Mito-tracker (red), and lysosome stained by Lyso-tracker (yellow); FITC-labeled NCs (green)). Scale bar, 20 μm. (E) Image-based colocalization analysis of NCs and organelles at indicated time points. R indicates the Pearson’s correlation coefficient of green fluorescence with organelle trackers’ fluorescence. Three images were used for triplicate analysis.

We next labeled CAT with Cy5.5 and SOD with FITC to visualize the partial release and cellular internalization of NCs from the C-cMAB/NCs. As shown in Figure S4B, NCs had better cellular internalization capability than other forms (free SOD or CAT), which agreed with the reported results.^19^ After treating the cells with cMAB/NCs and MMP-9 pretreated C-cMAB/NCs for 2 h, partial release and uptake of NCs could be observed, and the increasing signals were observed during the first 24 h and lasting until 48 h (Figure 4A and Figure S4C). Moreover, the red fluorescence of CAT showed strong colocalization with the green fluorescence of SOD during a 48 h incubation (Figure 4A,B), suggesting that internalized NCs did not undergo disassembly after internalization. We therefore explored the subcellular distribution of NCs within MLE-12 cells by labeling important intracellular organelles including mitochondria, lysosome, and nucleus (Figure 4C). As shown in Figure 4D, the overlap (yellow fluorescence) between the red fluorescence of Mito-Tracker and the green fluorescence of NC clearly indicated the preferable mitochondrial-targeting performance of NCs. This was attributed to the surface charge reversal ability of NCs in acidic lysosomes (Figure S5A), which kept in line with previous reports.^19^ As expected, the cells treated with cMAB/NCs, or MMP-9 pretreated C-cMAB/NCs also displayed enhanced yellow fluorescence, again demonstrating the active-targeting delivery of NCs from C-cMAB/NCs to the mitochondria after responding to MMP-9. Image-based colocalization analysis also supported this conclusion (Figure 4E).

### Effective mtROS Scavenging and Inhibition of AEC Damage *In Vitro*

**Figure 5:**
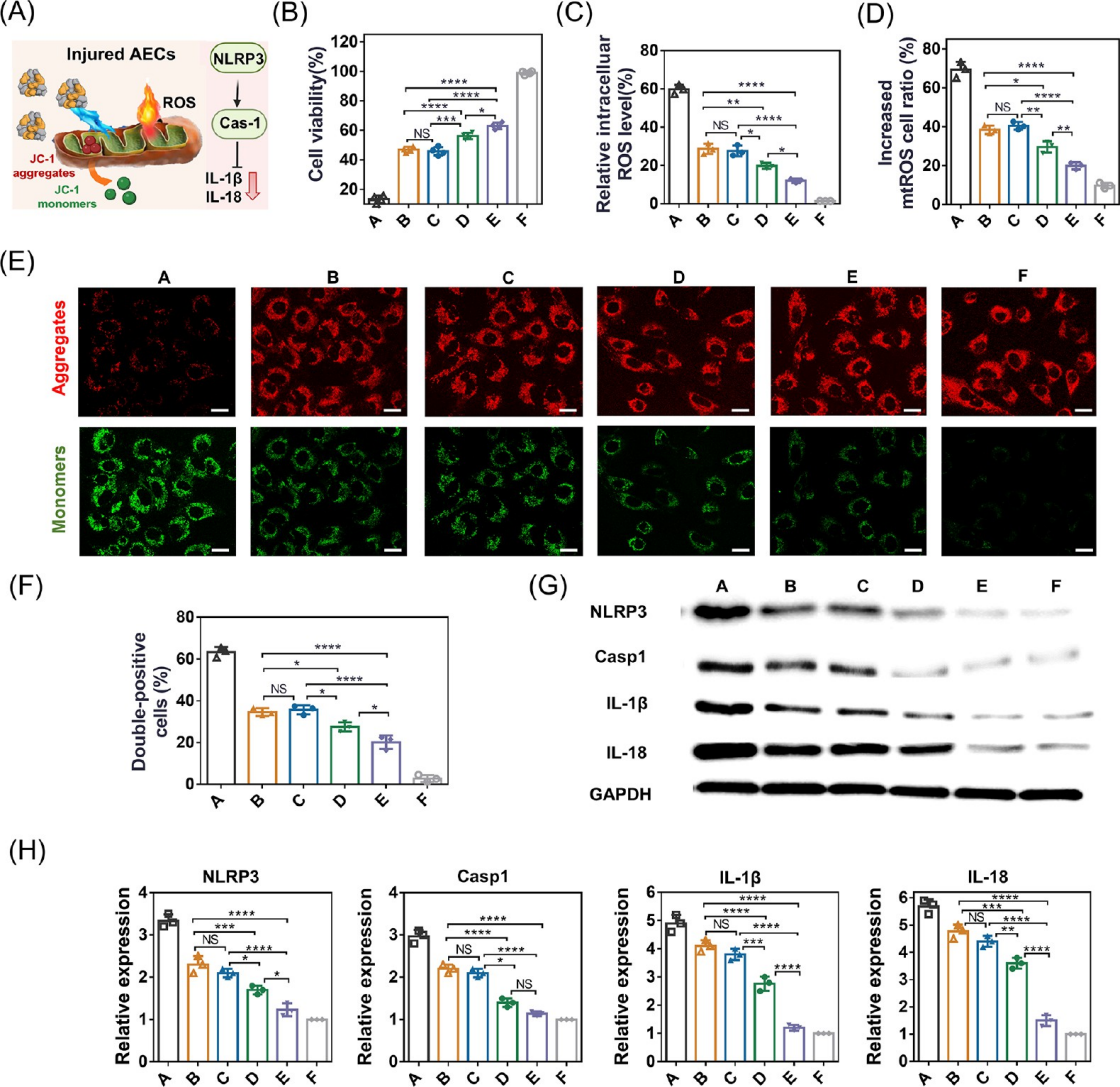
Effective elimination of mtROS and inhibition of cell damage by C-cMAB/NCs (created with BioRender.com). (A) Schematic illustration of protective mechanisms of C-cMAB/NCs in AECs against oxidative stress. (B–H) Cell viabilities (*n* = 4) (B), intracellular ROS level (C), mtROS level (D), JC-1 staining (E), quantitative analysis for the apoptosis (F) and (G, H) Western blotting of NLRP3, caspase-1, IL-1β, and IL-18 expression in MLE-12 cell after indicated treatments (*n* = 3). A, H_2_O_2_ + PBS; B, H_2_O_2_ + NCs; C, H_2_O_2_ + MP/NCs; D, H_2_O_2_ + cMAB/NCs; E, H_2_O_2_ + C-cMAB/NCs in the presence of MMP-9; and F, PBS. GAPDH was used as a housekeeping standard. scale bar, 20 μm. *p < 0.05, **p < 0.01, ***p < 0.001, ****p < 0.0001, n.s., not significant, *p* > 0.05.

Having confirmed the excellent mitochondria-targeting capability of C-cMAB/NCs, we then examined their epithelial protective efficacy (Figure 5A). Prior to *in vitro* epithelial cells protection studies, no significant cell cytotoxicity was observed after treating the cells with different formulations at a range of administered concentrations for 48 h (Figure S5B). All formulations showed a cell protective ability against oxidative stress via the CCK-8 assay (Figure 5B). Surprisingly, C-cMAB/NCs displayed efficient epithelial cell protective capacity against oxidative stress by targeted elimination of mtROS, as evidenced by much higher cell viability in H_2_O_2_-induced cells (Figure 5B) and significantly lower total cellular ROS levels (Figure 5C and Figure S5C) as well as the ROS levels in mitochondria (Figure 5D and Figure S5D). ROS-induced oxidative damage results in the severe disruption of the mitochondrial membrane. We therefore explored the impact of the C-cMAB/NCs on mitochondria function depending on the intracellular FI of JC-1.^24^ In the data reported herein, much less green fluorescence with significantly higher red fluorescence was produced in the cells by C-cMAB/NC treatment (in the presence of MMP-9), suggesting relatively healthy mitochondria relative to other treatments (Figure 5E and Figure S5E). As mitochondrial depolarization is a hallmark of cell apoptosis,^25^ we next conducted the Annexin V-FITC apoptosis assay. Consistent with previous results, C-cMAB/NC treatment significantly reduced the apoptotic cell percentages in MLE-12 cells, which were the lowest relative to those of other treatments (Figure 5F and Figure S5F). All of these factors highlighted the epithelial cell protective capacity of C-cMAB/NC treatment. Inflammasome activation plays prominent roles in cytokines induction, contributing to lung epithelial cell death after lung injury.^26^ Analysis of protein markers in MLE-12 cells indicated that expression levels of the pyroptosis- and inflammation-related markers including NLRP3, caspase-1, IL-1β, and IL-18 were significantly down-regulated in the C-cMAB/NC-treated group (in the presence of MMP-9) relative to the PBS group (Figure 5G,H). These results proved that C-cMAB/NCs, with high affinity to AECs and mitochondria-targeting capability, had excellent ability to scavenge mtROS, restore mitochondrial function, and inhibit cell apoptosis of epithelial cells by inhibiting inflammasome activation.

### Efficient Deposition in the Alveoli

**Figure 6:**
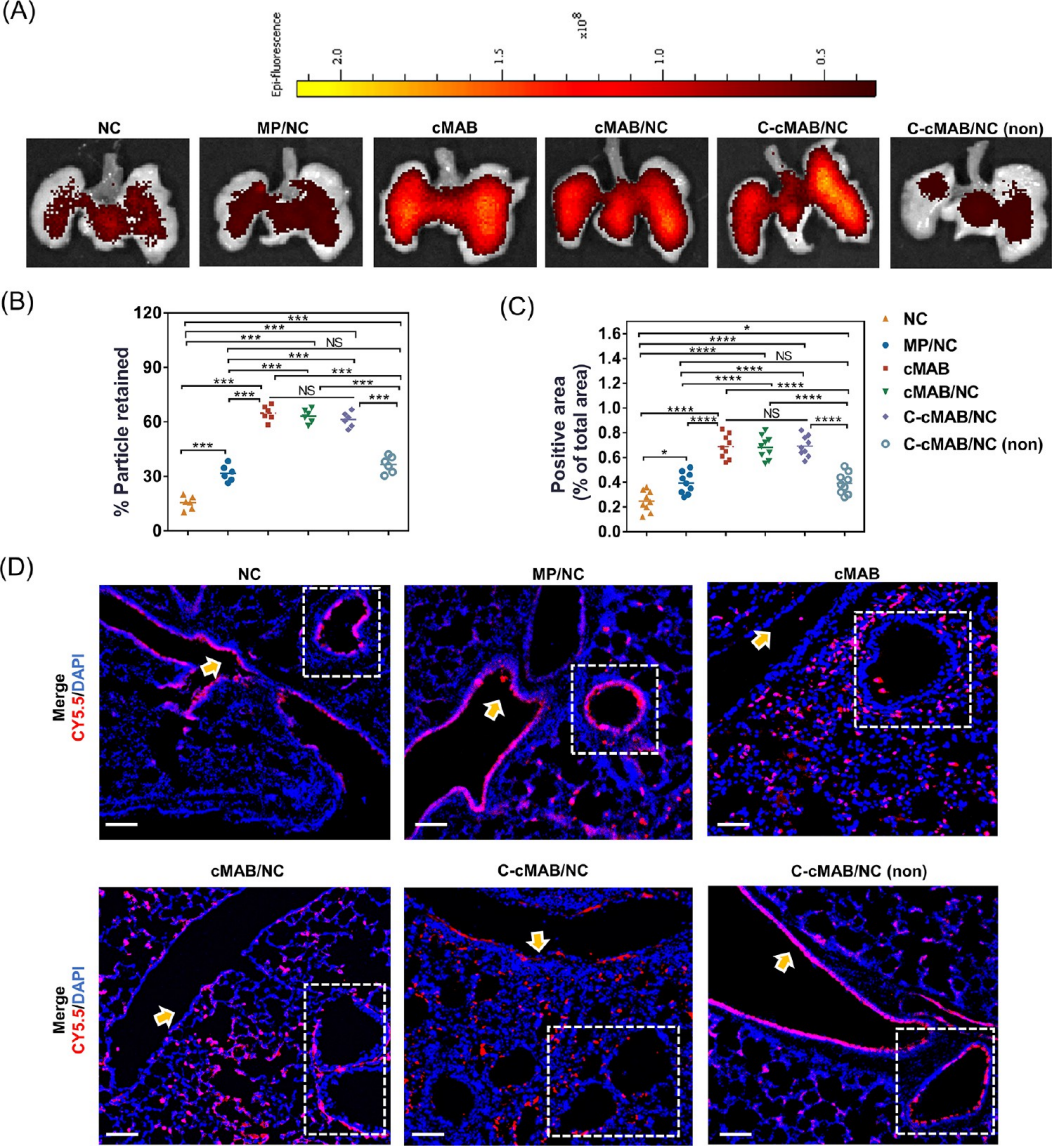
Efficient deposition into deeper alveoli following inhalation of C-cMAB/NCs. (A) IVIS imaging of lungs collected at 48 h after inhalation of Cy5.5-labeled NCs, MP/NCs, cMABs, cMAB/NCs, C-cMAB/NCs, and MMP-9 nonresponsive C-cMAB/NCs. (B) Retention of Cy5.5-labeled formulations in BAL fluid. (C) Image-based quantification of coverage of Cy5.5-labeled formulations in mouse left lungs. For each mouse, three images were used for a total of six measurements. (D) Localization of Cy5.5-labeled formulations in the large airway, small airway, and alveolar ducts (nuclei stained by Hoechst (blue) and Cy5.5-labeled formulations (red)). Arrows indicated formulations localization with a large airway; squares indicated formulation localization with a small airway. scale bar: 100 μm. *p < 0.05, ****p < 0.0001, n.s., not significant, *p* > 0.05.

Motivated by the cascade-targeting capability of C-cMAB/NCs *in vitro*, we further examined the biodistribution and fate of inhaled C-cMAB/NCs in LPS-treated hypoxic mice. IVIS imaging clearly revealed that compared with NC and MP/NC treatments, cMAB/NCs showed a relatively strong fluorescence signal located in the lung tissues, suggesting that the MABM coating enhanced retention (Figures 6A). In particular, C-cMAB/NCs exhibited an obviously strong fluorescence in lung tissues with a uniform particle distribution in each lobe, which reflected the better lung retention of our platform. However, in hypoxic LPS-challenged mice treated with MMP-9 nonresponsive C-cMAB/NCs, this effect was reversed. Additionally, the retention of C-cMAB/NCs via the lavage method (Figure 6B) were in line with IVIS results, consolidating the enhanced lung retention time as compared to conventional NCs or MP/NCs.

In agreement with *in vivo* retention results, NCs also failed to produce significant fluorescence in deeper alveoli at 48 h post intratracheal administration, which were mainly distributed among the large and small airways (Figure 6C,D; Figure S6). In contrast, lung tissues excised from mice treated with microformulations exhibited stronger red fluorescence in the alveoli, reflecting the satisfying particle aerodynamic diameter for efficient lung deposition. Note that cMAB/NCs and C-cMAB/NCs displayed efficient alveoli deposition with evidence of widespread fluorescence signal within alveoli, whereas MMP-9 nonresponsive C-cMAB/NC treatment did not show much benefit. These results substantiated that C-cMAB/NCs equipped with MMP-9 cleavable shells possessed superior alveoli deposition, making it beneficial for exerting a cascade-targeting property within the inflamed alveoli.

### C-cMAB/NCs Rescued the Monocytopenia in LPS-Treated Hypoxic Mice

**Figure 7:**
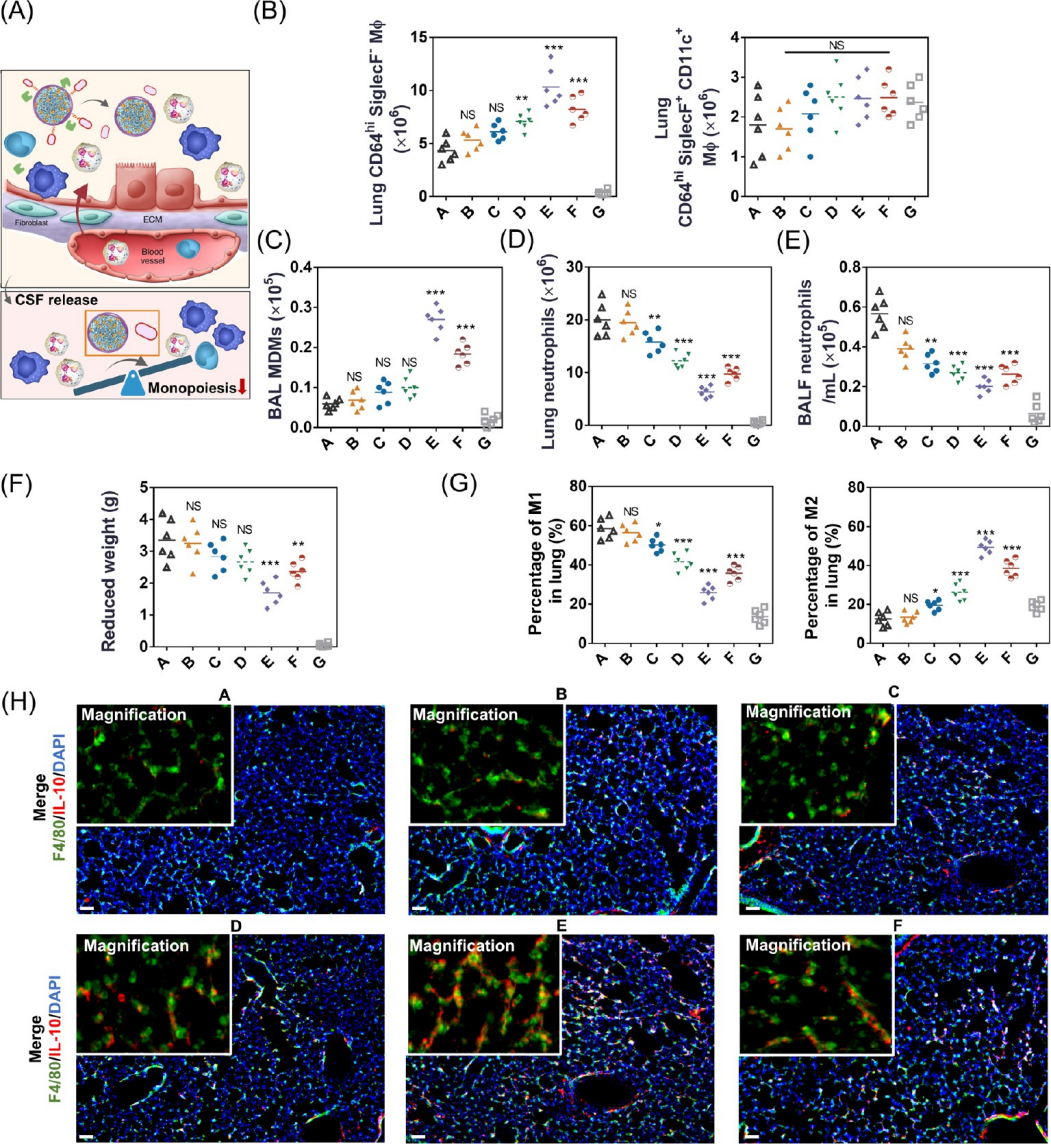
C-cMAB/NCs rescued the monocytopenia in LPS-treated hypoxic mice (created with BioRender.com and Depositphotos.com). (A) Schematic illustration of CSF release from C-cMAB/NCs in the hypoxic lung, contributing to the immune landscape reprogramming in lung injury. (B–H) CD64^hi^SiglecF^–^ macrophage, and CD64^hi^SiglecF^+^ CD11c^+^ macrophage numbers in the lung (B), BAL-recovered MDM numbers (C), neutrophil numbers in the lung (D), absolute numbers of neutrophils in the BAL (E), body-weight change (F), quantification analysis of CD80 (M1 macrophage biomarker) and CD206 (M2 macrophage biomarker) expressed on macrophages in the lung tissues (G), and representative IF images showing macrophages (F4/80) and IL-10 markers colocalization in the lung tissues (H) from LPS-challenged hypoxic mice treated with indicated formulations (*n* = 6). Nuclei were stained by Hoechst (blue), F4/80 (green), and IL-10 (red). scale bar, 100 μm; (inset) high-magnification image of F4/80^+^/IL-10^+^ in the lung tissues. A, hypoxic + LPS + PBS; B, hypoxic + LPS + NCs; C, hypoxic + LPS + MP/NCs; D, hypoxic + LPS + cMAB/NCs; E, hypoxic + LPS + C-cMAB/NCs; F, hypoxic + LPS + physical mixture of CSF and cMAB/NCs; G, sham control. **p < 0.01, ***p < 0.001, n.s., not significant, *p* > 0.05.

Hypoxia-induced lung innate immune cells changes have a sustained impact on inflammation resolution, such as persistent neutrophilic inflammation and epithelial cell apoptosis, two key poor prognostic features of ARDS.^27^ Encouraged by the potential of CSF-1 on reprogramming lung homeostasis,^13^ we then explored the impact of C-cMAB/NC treatment on inflammation outcomes in the LPS-treated hypoxic mice (Figure 7A and Figure S7). Monocyte recruitment and transformation into lung macrophages have been demonstrated as key factors driving inflammation resolution during ALI.^28^ Surprisingly, treatment with C-cMAB/NCs resulted in the most potent in enhancing the number of lung CD64^hi^SiglecF^–^ macrophages relative to PBS group (Figure 7B and Figure S8A), with significantly increased in BAL-recovered CD64^hi^SiglecF^–^ MDM numbers (Figure 7C and Figure S8B). In agreement with previous studies,^29^ no significant change in the number of CD64^hi^SiglecF^+^CD11c^+^ macrophages was observed, suggesting the importance of CSF grafting for monocytes and interstitial macrophages but not alveolar macrophages. Of note, the absolute number of Ly6G^+^CD11b^+^ neutrophils in the lung tissues (Figure 7D and Figure S8C) and BALF (Figure 7E) were both drastically reduced after the C-cMAB/NC treatment. In addition, the recovery of weight in C-cMAB/NCs, hypoxic, LPS-challenged mice again highlighted the efficacy of C-cMAB/NCs on rapidly reducing lung inflammation (Figure 7F). Based on these encouraging results, we further explored how C-cMAB/NC treatment-mediated expansion of CD64^hi^SiglecF^–^ macrophages promoted neutrophil clearance. Macrophages have been reported to show a high degree of plasticity depending on the environment, which can be simply classified as inflammatory M1 and pro-regenerative M2 phenotypes.^30^ In particular, previous studies have demonstrated that M2 macrophages facilitate alveolar repair in lung injury and inflammation resolution for lung immune homeostasis.^31^ Hence, we assessed the ratio of M2 phenotypes to the total number of macrophages via a flow cytometry analysis (Figure S8D). In the data reported herein, C-cMAB/NC treatment led to a significantly upregulation in lung pro-regenerative M2 macrophages, with about a 2.4-fold increase compared with their PBS counterparts, and reduced numbers of pro-inflammatory M1 macrophages in the lung tissues (Figure 7G and Figure S8D). More importantly, IL-10 have been confirmed as the key mediator of efferocytosis to alleviate neutrophils-derived secondary damage after injury.^32^ Consistent with these roles, C-cMAB/NC treatment resulted in significantly improved IL-10 levels in both the serum and BAL from LPS-treated hypoxic mice relative to the PBS group (Figure S8E). In contrast, other treatments did not show much benefit. IF staining images further consolidated that inhalation of C-cMAB/NCs outperformed other treatments in improving the numbers of IL-10-producing macrophages in lung tissues (Figure 7H). Therefore, all of these factors could create a pro-regenerative environment after lung injury, which were determinant for driving hypoxia-mediated inflammation resolution.^33^

### Therapeutic Efficacy in LPS-Treated Hypoxic Mice

**Figure 8:**
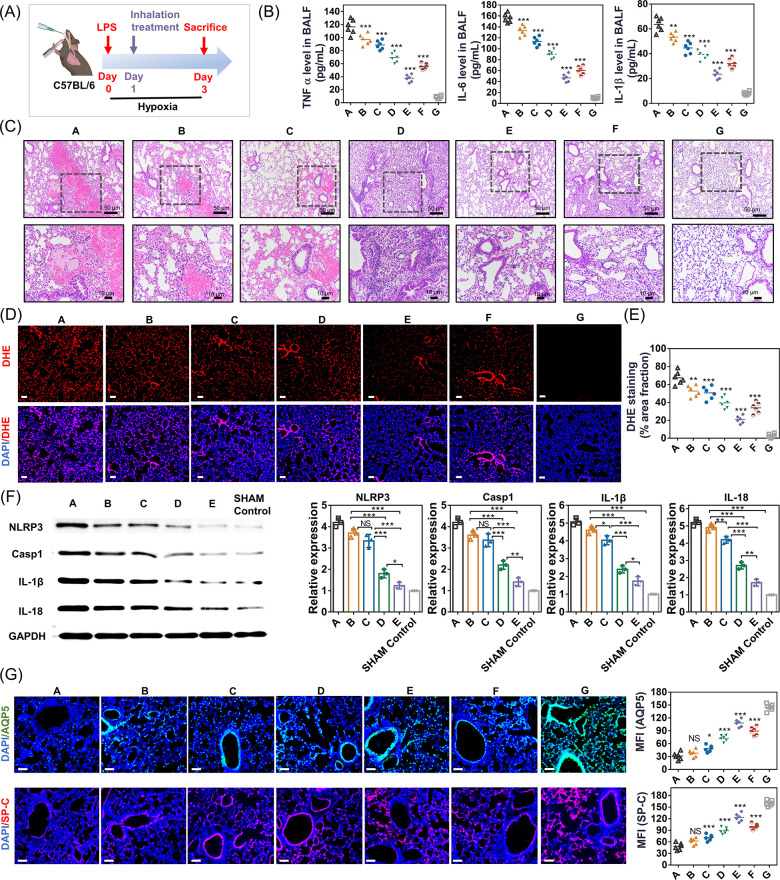
Inhalation of C-cMAB/NCs inhibited the lung hyper-inflammatory conditions and alleviated the inflammasome-mediated AECs damage in LPS-treated hypoxic mice. (A) Schematic illustration of C-cMAB/NC treatment. (B–H) Pro-inflammatory cytokines including TNF α, IL-6, and IL-1β secretion in BALF (B), H&E staining images (C), representative DHE staining of lung tissues (D), image-based DHE fluorescence signal analysis (E), Western blotting of NLRP3, caspase-1, IL-1β, and IL-18 expression in lung tissue homogenates. GAPDH was used as a housekeeping standard (F); representative IF images and quantification of AQP5 and SP-C expression in lung tissues (G) forming LPS-challenged hypoxic mice after indicated treatments (*n* = 6). Nuclei stained by Hoechst (blue), AQP5 (green), DHE dye, or SP-C (red). Scale bar, 100 μm. For each mouse, three images were used for a total of six measurements. A, hypoxic + LPS + PBS; B, hypoxic + LPS + NCs; C, hypoxic + LPS + MP/NCs; D, hypoxic + LPS + cMAB/NCs; E, hypoxic + LPS + C-cMAB/NCs; F, hypoxic + LPS + physical mixture of CSF and cMAB/NCs; G, sham control. *p < 0.05, **p < 0.01, ***p < 0.001, n.s., not significant, *p* > 0.05.

Finally, we tested the therapeutic efficacy of C-cMAB/NC treatment in a well-established mouse model of HALI. The experiment lasted 3 days, and the treatment was conducted on the first day after LPS induction (Figure 8A). Hypoxic LPS-induced hyper-inflammation and the resultant lung tissues damage are two primary causes of mortality.^34^ Therefore, we collected the BALF from LPS-treated hypoxic mice treated with C-cMAB/NCs at the study end point for cytokine measurement. Note that inhalation of C-cMAB/NCs significantly inhibited hyper-inflammatory conditions as shown by sharp decreases in the TNF-α, IL-6, and IL-1β as well as total protein levels in the BALF (Figure 8B and Figure S9A). To determine the key elements of C-cMAB/NC responsible for its anti-inflammatory effect, the BALF from mice treated with free SOD, CAT, TA, CSF, or blank carriers including cMAB and C-cMAB was collected. Results clearly indicated the encouraging effects of the C-cMAB carrier on the suppression of inflammatory cytokines (Figure S9B). As a result of inflammatory inhibition, the survival rate of C-cMAB-treated mice was extended by 80% at 3 days (Figure S9C). Based on these results, we further investigated their ability to alleviate lung tissue damage. Histological examination by H&E staining of the left lung tissues demonstrated that C-cMAB/NC treatment resulted in a substantial reduction in pulmonary edema, alveolar wall incrassation, and alveolar inflammatory cell infiltration (Figure 8C). Additionally, a marked decrease in the wet/dry lung weight ratio (Figure S9D), a key indicator of pulmonary edema, was observed in mice receiving C-cMAB/NC treatment, again demonstrating their strong therapeutic benefit to the lung tissues. Motivated by the incomparable mtROS scavenging efficiency of C-cMAB/NCs as shown by the aforementioned *in vitro* studies, we then evaluated their antioxidant effect on lung epithelial barrier integrity. As expected, DHE staining of excised lung tissues verified the effective ROS scavenging capacity of C-cMAB/NCs as confirmed by the lowest ROS accumulation in the lung (Figure 8D,E; Figure S9E). In addition, the expression of the pyroptosis- and inflammasome-activation related proteins including NLRP3, caspase-1, IL-1β, and IL-18 were the lowest in mice receiving C-cMAB/NCs (Figure 8F). Based on these encouraging results, we further examined the effect of C-cMAB/NCs on alveolar repair. At the end point of administration, we evaluated the distribution of AEC1 and AEC2 in lung tissues, which are the key indicators responsible for the alveolar integrity.^35^ As expected, inhalation of C-cMAB/NCs significantly promoted the levels of AQP5 (an AEC1 marker) and SP-C (an AEC2 marker) in the lung tissues (Figure 8G and Figure S9F) relative to those of other treatments, again demonstrating their strong lung protective effect. After 2 days of treatment, a single intratracheal injection of C-cMAB/NCs was able to rescue 100% of the mice (Figure S9C), which was significantly more than that of PBS (70%), NCs (80%), or MP/NCs (90%). Collectively, these findings proved that inhalation of C-cMAB/NCs effectively alleviated the lung hyper-inflammatory conditions and simultaneously inhibited inflammasome-mediated AECs damage in LPS-treated hypoxic mice.

## Conclusions

ARDS is a complex and dynamic disorder that is extremely challenging to treat by targeting single mediators or pathogenic pathways. Here, we developed an inhalable core–shell intelligent platform (C-cMAB/NC) to address multiple pathogenic factors in ALI by multiple-therapeutic loading, efficient lung deposition, high affinity to AECs, and mitochondria targeting. This drug/drug as weeding and uprooting strategy offers an appealing complement to conventional anti-inflammatory treatments, supported by addressing the nonspecific tissue/cell uptake for clinically used chemotherapeutics and directly creating a pro-regenerative microenvironment leading to pulmonary functional recovery. Note that nano-SOD/CAT actioned as the “weeding” part of the strategy, ensuring efficient inhibition of inflammasome-mediated AEC damage by reversing mitochondrial depolarization. The other drug (CSF) served as the “uprooting” part of the strategy by rapidly expanding MDM numbers in the hypoxic lung, contributing to macrophage polarization into pro-regenerative M2 phenotype and a concomitant increase in anti-inflammatory IL-10 production. As a result, C-cMAB/NC treatment created a pro-regenerative microenvironment in the hypoxic lung, with down-regulation of critical pro-inflammatory cytokines and suppression of neutrophil-associated inflammation, enabling resolution of lung injury and inflammation. In addition, the fabrication of multiple therapeutics–loaded MPs (C-cMAB/NCs) was facile and efficient via physically encapsulated nanosized NCs and chemically conjugated CSF chains. Looking toward the future, we believe that our strategy of attenuating multiple pathogenic factors is a promising intervention, which could be used in conjunction with supportive care to improve survival of ARDS. Furthermore, this inhalable biomimetic microparticle platform may have application to other inflammatory ling diseases.

## Materials and Methods

### Macrophage Apoptotic Body Membrane Isolation and Characterization

RAW 264.7 cells were treated with LPS at 1 μg/mL for 24 h to induce apoptosis *in vitro*.^36^ The culture media were thereafter collected, double centrifuged at 50*g* for 5 min, and single centrifuged at 1000*g* for 10 min. The resulting MAB pellets were washed with PBS and quantified using a bicinchoninic acid (BCA) protein assay kit (Beyotime, Beijing, China). The identification of MABs were conducted using an Annexin V-FITC apoptosis detection kit (Sigma-Aldrich, St. Louis, MO) and antimouse C1q antibody. To prepare MABM vesicles, collected MABs were exposed to hypotonic solution (0.25 × PBS) at 4 °C for 2 h with gentle sonication for 5 s. After centrifugation at 100*g* for 10 min and at 10,000*g* for 10 min, MABM pellets were washed three times with water and collected for further use. The morphology and size of MABs and MABMs were determined by scanning electron microscopy (SEM, S-4700, Hitachi, Japan) and dynamic light scattering (DLS, Zetasizer Nano ZS90, Malvern, UK), respectively. Western blotting analysis was conducted to reveal the protein components of the MABs and MABMs.

### Preparation of MABM Camouflaged Nanocomplex-in-PLGA Microparticles (cMAB/NCs)

First, SOD/CAT NCs were prepared by gently mixing of tannic acid (TA) solution (200 μL, 2.9 mM) with SOD and CAT mixed solution (100 μL, 5 mg/mL for each enzyme) in PBS (4.2 mL) for 10 min and adjusting the pH of the mixture to 7.0.^19^ NCs were thereafter encapsulated into MPs by an adjusted water/oil/water double emulsion solvent evaporation method.^37^ Briefly, PLGA (100 mg) was fully dissolved in dichloromethane (2 mL) containing PVP-K12 (25 mg) as the organic phase. Meanwhile, freshly prepared NCs (10 mg/mL) were added to PBS (2 mL) as the first aqueous phase. The NC dispersion was then added dropwise into the organic phase, followed by sonication via an ultrasonic probe for 2 s. Afterward, the resulting emulsion was injected into the second aqueous solution (6 mL of PVA solution with 6% (w/v)), vigorously vortexed for 15 s, and dropwise added to of PVA solution (8 mL, 1.5% (w/v)). After being stirred for 4 h, the MP/NCs were obtained via centrifugation at 3000*g* followed by washing three times with water. Next, the MABM camouflaged MP/NCs were fabricated by mixing the MP/NCs suspension with the MABM suspension at a ratio of 2:1 (w/w) for 1 h under vortex stirring and then sonication in an ultrasonic bath for 5 min. The obtained cMAB/NCs were subjected to freeze-drying for further use.

### Fabrication of CSF-Grafted cMAB/NCs (C-cMAB/NCs)

A three-step reaction was proposed to conjugate CSF chains on the cMAB/NC surface. First, DBCO–CSFs were prepared via a classical amidation reaction. Briefly, DBCO-NHS was reacted with a PBS solution of CSF at a ratio of 2:1 (w/w) for 4 h under continuous stirring. Second, MMP-9 responsive peptides were introduced to the cMAB/NC surface by cross-linking the amine-containing MABM coating with sulfydryl-functionalized polypeptides according to our previous report.^31^ Next, C-cMAB/NCs were prepared by reagent-free click reaction of the N3 group of peptide linkers with the DBCO group of the CSF chains. Briefly, DBCO–CSFs were prepurified by ultrafiltration (100-kDa, Millipore) and then reacted with the resulting MMP-9 responsive cMAB/NC solution at 37 °C for 30 min. Following centrifugation at 164,000*g* for 40 min and washing three times with PBS, the resulting C-cMAB/NCs were subjected to freeze-drying for further use.

### Physicochemical Properties Characterization

NC size and zeta potential were determined by DLS (Zetasizer Nano ZS90, Malvern, UK), and its morphology was determined by a transmission electron microscope (TEM, JEM-2100F, Tokyo, Japan). The ROS scavenging activity of NCs was determined using a total superoxide dismutase assay kit (Beyotime, Beijing, China) according to the manufacturer’s protocol. For micro-sized formulations including PLGA MPs, MP/NCs, cMAB/NCs, and C-cMAB/NCs, their geometric size and particle size distribution were measured by laser diffraction (Sympatec, HELOS/KP, Germany), and morphology was visualized by SEM (S-4700, Hitachi, Japan). To quantify the porosity on the surface of MP/NCs after MABM coating, a quotient of pore areas to total surface areas of particles (gray/white) on their SEM images was calculated via a macroinstruction programmed for ImageJ. To further reveal a successful membrane coating, fluorescently labeled cMAB/NCs were fabricated using PLGA-FITC and Cy5.5-labeled MABMs during synthesis. Then, the resulting cMAB/NCs were resuspended with glycerol for visualization with a confocal microscope (CLSM; Leica SP8, Germany). The Coomassie blue staining and Western blotting analysis were conducted to explore protein profiles of cMAB/NCs. To further confirm the membrane coating percentage, Cy5.5labeled MABMs were prepared by incorporating Cy5.5 (0.1% (w/w)) into the MABM vesicles as previously reported.^38^ After that, the fluorescence intensity (FI) in the MABM suspension before fusion and in the supernatant after the cMAB/NC concentration was separately assessed via a microplate reader (excitation at 683 nm and emission at 703 nm; PerkinElmer, USA) and calculated according to the following equation.
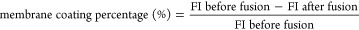
1

Since the formulations contained two different enzymes, we first determined that there is no interference in activity of SOD to CAT and vice versa when both were assayed using the respective kits (Sigma-Aldrich, St. Louis, MO). Note that upon assay of the SOD activity in the formulations, the control group (no xanthine oxidase) was included for reduction of TA interference as the manufacturer’s protocol.^19^ The encapsulation efficiency (EE) for each enzyme (SOD or CAT) in the NCs, PLGA MPs, and cMABs was determined from the difference in the amount of each enzyme added and the amount that was detected in the supernatant collected following centrifugation, as described above under the formulation protocol. Additionally, the EE of CSF in C-cMAB/NCs was calculated from the difference in the amount of CSF added to the formulation and the amount assayed in the supernatant following centrifugation under the formulation protocol via a M-CSF ELISA kit (Sigma-Aldrich, St. Louis, MO). The storage stability of C-cMAB/NCs was determined by measuring the CSF cargo leakage after storage at 4 °C for 7 days using the corresponding kit.

### MMP-9 Triggered CSF Release from C-cMAB/NCs

To determine the enzyme responsive release property of CSF from C-cMAB/NCs, 2 mL of C-cMAB/NCs (1 mg/mL) was dispersed in PBS (10 mL, 150 mM, pH 7.4) containing Tween-80 (0.05% (w/v)) and various concentrations (0, 5, 25, 50 nM) of MMP-9 on a shaker rotating at 37 °C and 120 rpm.^39^ At each time interval, 200 μL of samples was withdrawn and centrifuged at 620*g* for 10 min to assay CSF content in the supernatant using the corresponding ELISA kit (Sigma-Aldrich, St. Louis, MO).

### *In Vitro* Aerodynamic Performance

The *in vitro* aerodynamic performance of the microformulation was evaluated using a next generation impactor (NGI, Copley Scientific, UK) following the procedure detailed in the US Pharmacopoeia.^40^ Before measurement, the USP throat and all the stages of the NGI were coated with the Tween 20/ethanol solution (2% w/v) to minimize particle bouncing. Accurately weighed powders (20 mg) of MP/NCs, cMAB/NCs, and C-cMAB/NCs were loaded into individual HPMC capsules (no. 3, Suzhou Capsugel Ltd., China), which was further placed in the sample compartment of a Cyclohaler apparatus, connected to the inlet of an NGI instrument. Upon actuation, the capsule was pricked to release the powder, which was delivered into the NGI at a flow rate of 100 L/min. Powder from each NGI stages were collected for further analysis. To determine the NC amount, fluorescently labeled MP/NCs, cMAB/NCs, and C-cMAB/NCs were fabricated using Cy5.5-labeled CAT during synthesis.^41^ After collection, the powder was dissolved in NaOH solution (1N, 1 mL) independently, and the amount of loading NCs was determined based on a calibration curve established by plotting the fluorescence versus the concentration of free Cy5.5-CAT. Experimental mass median aerodynamic diameter (MMAD_e_) was determined from the analysis of the NGI data.

### Interaction of Particles with Cells

To explore the interaction of MABM coated MPs, primary AMs, neutrophils, and MLE-12 cells were seeded in 35 mm glass bottom culture dishes (2 × 10^5^ cells/mL), respectively. Meantime, fluorescently labeled NCs, MP/NCs, cMAB/NCs, and C-cMAB/NCs were fabricated using Cy5.5-labeled CAT during synthesis. Cy5.5-labeled cMABs were then fabricated using Cy5.5-labeled MABM coating. Afterward, the cells were treated with different formulations at equivalent amounts of SOD/CAT enzymes in saline (SOD = 176 μg, CAT = 236 μg) and incubated for 2 h. After washing three times with cold PBS, the cells were collected for flow cytometry analysis (BD Biosciences, USA) and imaging by a confocal microscope (Leica SP8, Germany). Prior to observation, cell nuclei were stained with DAPI.

### Intracellular NC Release and Subcellular Localization

MLE-12 cells were seeded in 35 mm glass bottom culture dishes (2 × 10^5^ cells/mL). Meantime, the fluorescently labeled NCs, cMAB/NCs, and C-cMAB/NCs were fabricated using Cy5.5-labeled CAT and FITC labeled SOD during synthesis. Afterward, the cells were incubated with the fluorescently labeled NCs, cMAB/NCs, and C-cMAB/NCs at equivalent amounts of SOD/CAT enzymes in saline (SOD = 176 μg, CAT = 236 μg) and imaged using a confocal microscope (Leica SP8, Germany) at 2, 4, 24, and 48 h, respectively. For further investigating the cMAB carrier distribution, Cy5.5-labeled MABMs were used to camouflage MP/NCs and incubated with the cells for imaging.

For subcellular localization analysis, the cells were cultured on culture dishes (2 × 10^5^ cells/mL) and further incubated with NCs, cMAB/NCs and C-cMAB/NCs at equivalent amounts of SOD/CAT enzymes in saline (SOD = 176 μg, CAT = 236 μg) for 24 h. After incubation, the cells were washed with cold PBS, stained by Mito-tracker, Lyso-tracker, and DAPI, as per the manufacturer’s protocol for imaging via a confocal microscope. The colocalization values were calculated via ImageJ software.

### *In Vitro* Treatment against Oxidative Damage in MLE-12 Cells

The MLE-12 cells with oxidative damage were obtained by H_2_O_2_ induction (500 μM).^13^ First, the protective efficacy of C-cMAB/NCs against H_2_O_2_-induced oxidative stress was evaluated based on a CCK-8 assay. Briefly, the cells were seeded into 96-well plates (5 × 10^3^ cells/well) and treated with NCs, MP/NCs, cMAB/NCs, and C-cMAB/NCs at equivalent amounts of SOD/CAT enzymes in saline (SOD = 176 μg, CAT = 236 μg) for 12 h before H_2_O_2_ induction for additional 24 h. For comparison, the MMP-9 nonresponsive C-cMAB/NCs were fabricated using a polypeptide linker with D-type amino acids. Next, the cells were seeded into 12-well plates (2 × 10^5^ cells/well) and treated with NCs, MP/NCs, cMAB/NCs, or C-cMAB/NCs at equivalent amounts of SOD/CAT enzymes in saline (SOD = 176 μg, CAT = 236 μg). After a 12 h incubation, the cells were then incubated with H_2_O_2_ for an additional 24 h. Then, the cells were washed with cold PBS and collected for analysis. The intracellular total ROS and mitochondrial ROS production levels, as well as the mitochondrial membrane potentials, were measured using fluorescent dye DCFH-DA, MitoSOX, or JC-1, as per the manufacturer’s protocol, respectively. Data were required by a confocal microscope (Leica SP8, Germany) or flow cytometry (BD Biosciences, USA). Cell apoptosis was determined using an Annexin V-FITC apoptosis detection kit (Sigma-Aldrich, St. Louis, MO), and data were required by flow cytometry (BD Biosciences, USA). Western blotting analysis was used to determine the expression of the pyroptosis- and inflammation-related protein markers including NLRP3, caspase-1, IL-1β, and IL-18 in the cells treated with different formulations and the resulting blots were quantified by ImageJ software.

### Mouse Models of Hypoxic Acute Lung Injury

Mice were anesthetized, treated with LPS (50 μL, 4 mg/kg) via intratracheal instillation, and then exposed to hypoxia conditions (10% O_2_) immediately thereafter for up to 3 days.^13^ At 24 h post LPS instillation, mice were treated with indicated formulations by intrathecal injection via a dry powder insufflators model (YAN 30010). For the *in vivo* cell-targeting and biodistribution test, Cy5.5-labeled NCs, MP/NCs, cMABs, cMAB/NCs, and C-cMAB/NCs (equivalent amounts of SOD/CAT enzymes in saline (SOD = 4400 μg/kg, CAT = 5900 μg/kg)) were fabricated. For *in vivo* therapeutic evaluation, mice were treated with NCs, MP/NCs, cMAB/NCs, and C-cMAB/NCs (equivalent amounts of SOD/CAT enzymes in saline (SOD = 4400 μg/kg, CAT = 5900 μg/kg)) or physical mixture of cMAB/NCs and free CSF (0.2 mg/kg), prior to cull on day 3.

### *In Vivo* Biodistribution Study

Retention of inhaled formulations in the lungs was studied by two complementary methods: the whole-lung method and the lavage method.^42^ For the whole lung method, 48 h after inhalation of different formulations, mice were sacrificed, and the lung tissues were harvested for imaging on an IVIS system (PerkinElmer, USA) and cryosections, respectively. For the lavage method, similarly treated mice were sacrificed, and the bronchoalveolar lavage (BAL) supernatants was collected by lavaging the lungs three times with 0.8 mL of cold PBS. Fluorescently labeled particles in the BALF were measured via a microplate reader (excitation at 683 nm, emission at 703 nm; PerkinElmer, USA).

To investigate the *in vivo* distribution of inhaled C-cMAB/NCs, the lung frozen slices were stained with the cell nucleus (DAPI). Finally, the resulting slices were observed with a fluorescent microscope (DMi8, Leica, Germany) and the particle distribution was quantified using ImageJ software.

### *In Vivo* Cell-Targeting Study

Forty-eight h after inhalation of different formulations, lung tissues were collected to observe the majority cell subtype colocalized with fluorescent labeled MPs. The lung tissues were thereafter washed with cold PBS, minced, and dissociated by incubating with a working solution (5 mL of RPMI containing 1.5 mg/mL collagenase I, 0.625 mg/mL collagenase D, and 50 U/mL DNase I) for 45 min at 37 °C and 150 rpm. Afterward, digested lung tissues were passed through a 70 μm cell strainer, treated with ACK lysing buffer for 5 min, and pelleted at 300 g for 5 min. The samples were redispersed in PBS containing FBS (2% (v/v)) and then filtered through a 40 μm strainer to obtain single-cell suspensions for flow cytometry analysis.

### Lung and Alveolar Cell Sampling and Flow Cytometry Analysis

Forty-eight h after inhalation of different formulations, BAL cells were collected as previously mentioned. After perfusion, the lung tissues were washed and harvested for enzymatic dissociation to obtain single-cell suspensions. Cells from BALF and lung tissues were treated with ACK lysing buffer and counted for flow cytometry analysis.^43^ Before staining with antibodies, mouse cells were treated with Fc blocking (1:1000). For the *in vivo* cell-targeting study, cells were stained with antibodies against immune, epithelial, and endothelial cell markers. For the *in vivo* efficacy study, cells were stained with antibodies against macrophage, monocyte-derived macrophage (MDM), and neutrophil cell markers. Cells were acquired on the LSRFortessa (BD Biosciences, USA), and data were analyzed using FlowJo software.

### BAL Cytokine and Total Protein Quantification

On day 3, the treatments were terminated, and BAL supernatants were collected for evaluating the total protein and pro-inflammatory cytokines including TNF-α, IL-6, and IL-1β levels using BCA and corresponding ELISA kits (BioLegend, San Diego, USA), as per the manufacturer’s instructions, respectively.

### Lung Histology and Immunofluorescence

Forty-eight h after inhalation of different formulations, lung tissues were collected for H&E and IF staining. Paraffin-embedded lung tissues were cut into 5 μm-thick sections and stained with H&E. Lung tissues frozen sliced were cut into 18 μm-thick sections, incubated with primary antibodies (anti-IL-10, anti-F4/80, AQP5 or SP-C) at 4 °C overnight, and then incubated with the corresponding secondary antibody (AlexaFluor 488 goat antirabbit IgG H&L or AlexaFluor 594 goat antimouse IgG H&L) for 2 h at room temperature. To access ROS production in the hypoxic lung, frozen sections of lung tissues were directly treatment with DHE solution (5 μmol/L) for 15 min at room temperature. Prior to observation, cell nuclei were stained with DAPI. Fluorescence imaging was conducted via a fluorescent microscope (DMi8, Leica, Germany) and quantitative analysis using ImageJ software.

### Lung Injury Measurements and Western Blotting

The body weight of mice was individually recorded every day, and the survival of different treatment groups was analyzed during the experiment period. On day 3, mice were sacrificed, and the lung tissues were collected, washed, and weighed as “wet” weight. Subsequently, the lung tissues were placed at 80 °C for 72 h for recording “dry” weight, and then the wet/dry weight ratio was calculated. For the Western blot assay, lung tissue samples from different formulations treated, hypoxic, and LPS-challenged mice were lysed with RIPA lysis buffer containing phenylmethanesulfonyl fluoride. An equal protein amount of each sample was separated by SDS–PAGE and then transferred onto PVDF membranes. After blocking, the membranes were separately incubated with primary antibodies (NLRP3, caspase-1, IL-1β, or IL-18) at 4 °C overnight and then treated with the corresponding secondary antibody at room temperature. Finally, the membranes were imaged by a chemiluminescence system (Bio-Rad, USA) and quantified by ImageJ software.

### Statistics

Statistical analysis was conducted using Prism 7 software (Graph Pad). Data were presented as the mean ± standard deviation. Unpaired Student’s test was utilized to compare two groups, while one-way ANOVA with Tukey’s comparisons tests was used to compare multiple groups. *p* values less than 0.05 were considered statistically significant.

## Supporting Information Available

Materials, cell culture, and animals; physicochemical properties of microformulations; represented morphology images of isolated primary AMs and neutrophils, and cellular uptake after incubation with indicated treatments; FACS gating strategy used to identify subtypes of cells from lung tissues and represented flow cytometry analysis of different treatment groups distribution in different cells; confocal images of MLE-12 cells taken for qualitative NCs release; quantitative analysis of the intracellular ROS level, mtROS level, and apoptosis of MLE-12 cells after treatment with different formulations; localization of Cy5.5-labeled formulations in the mouse left lungs; establishment of LPS-treated hypoxic mouse models; FACS gating strategy used to identify lung macrophages and neutrophils and quantification analysis of CD80 and CD206 on macrophages as well as the IL-10 level in BALF after different treatment groups; inhalation of C-cMAB/NC alleviated lung pathological disorders in mice with hypoxic ALI; and MMAD_t_ as well as drug loading of microparticles; and a list of abbreviations (PDF)
