# Supplementary material for: Inhaled Macrophage Apoptotic Bodies-Engineered Microparticle Enabling Construction of Pro-Regenerative Microenvironment to Fight Hypoxic Lung Injury in Mice
Source: ACS Nano. 2024 May 10;18(20):13361–76. doi: 10.1021/acsnano.4c03421 (PMC11112977; doi:10.1021/acsnano.4c03421)
Supplement: Supplementary file 1 — nn4c03421_si_001.pdf [file nn4c03421_si_001.pdf]

Supplemental information

Inhaled Macrophage Apoptotic Bodies-Engineered Microparticle Enabling Construction of Pro-Regenerative Microenvironment to Fight Hypoxic Lung Injury in Mice

Chang Liu [1], Xingping Quan [1], Xidong Tian [1], Yonghua Zhao [1,2], Hai-Feng Li [3], Judith Choi Wo Mak [4], Zhenping Wang [5], Shirui Mao [6,7], Ying Zheng [1,2]*

[1] *State Key Laboratory of Quality Research in Chinese Medicine, Institute of Chinese Medical Sciences, University of Macau, Macau 999078, China*

[2] *Department of Pharmaceutical Sciences, Faculty of Health Sciences, University of Macau, Macau 999078, China*

[3] *Joint Key Laboratory of the Ministry of Education, Institute of Applied Physics and Materials Engineering, University of Macau, Macau 999078, China*

[4] *Department of Pharmacology and Pharmacy, LKS Faculty of Medicine, The University of Hong Kong, Hong Kong 999077, China*

[5] *Department of Dermatology, School of Medicine, University of California, San Diego, CA 92093, USA*

[6] *School of Pharmacy, Shenyang Pharmaceutical University, Shenyang 110016, China*

[7] *Joint International Research Laboratory of Intelligent Drug Delivery Systems, Ministry of Education, China*

*Corresponding author. Tel.: +853 88224687; fax: +853 28841358 (Ying Zheng).

E-mail address: yzheng@um.edu.mo (Ying Zheng).

Supplemental Experimental Section

Materials

PLGA (Resomer® RG 503H, lactide:glycolide ratio in 50:50) was obtained from Boehringer Ingelheim (Germany). PLGA-FITC, Sulfosuccinimidyl 4-(N-maleimidomethyl) cyclohexane-1-carboxylate (Sulfo-SMCC) and Dibenzocyclooctynesulfo-N-hydroxysuccinimidyl ester (DBCO-sulfo-NHS) were acquired from Ruixi Technology Co. Ltd. (Xian, China). 5-azidopentanoic acid-GPLGLAGQC polypeptide were obtained from Beijing Scilight Biotechnology Ltd (Beijing, China). Poly (vinyl alcohol) (PVA 205), Polyvinyl pyrrolidone K-12 (PVP K-12) purchased from Macklin (Beijing, China). Macrophage colony-stimulating factor (CSF-1) were acquired from Thermo Fisher Scientific (Massachusetts, USA). Tannic acid, superoxide dismutase, catalase, LPS (Escherichia coli 0111: B4), mucin (Type II, from porcine stomach), salmon deoxyribonucleic acid (DNA), egg yolk emulsion, diethylenetriaminepentaacetic acid (DTPA), DNase I, Collagenase I and Collagenase D were purchased from Sigma-Aldrich (Massachusetts, United States). Ammonium-Chloride-Potassium (ACK) lysing buffer and Diff-Quik Staining were obtained from Solarbio Biotechnology Co. Ltd. (Beijing, China). All other chemical reagents were of analytical or chromatographic grade. Fluorescence probes DAPI, Diacetyldichlorofluorescein (DCFH-DA), MitoSOX, JC-1, dihydroethidium (DHE), FITC, Cy5.5 were purchased from Invitrogen (Carlsbad, USA). Monoclonal antibodies including CD45 (30-F11), CD11b (M1/70), Gr-1 (RB6-8C5), CD326 (Ep-CAM) (9C4), CD64 (X54-5/7.1), CD11c (N418), Ly6C (HK1.4), SiglecF (E50-2440), CD 31 (390), F4/80 (SP115) and CD24 (ML5) were obtained from BioLegend (San Diego, USA). Anti-mouse CD32/CD16 (Fc-block) were obtained from BD Biosciences (San Diego, USA). Anti-NLRP3 antibody (EPR23094-1), Anti-IL-18 antibody (polyclonal), Anti-Aquaporin 5 antibody (polyclonal), and second antibodies including Goat anti-mouse IgG H&L (AlexaFluor 594) and Goat anti-rabbit IgG H&L (AlexaFluor 488) were achieved from Abcam (California, USA). Cleaved Caspase-3 Antibody (Asp175), Integrin β1 Antibody (polyclonal), Caspase-1 Antibody (polyclonal), IL-1β Mouse mAb (3A6) was purchased from Cell Signaling Technology (Danvers, MA). IL-10 Antibody (A-2) was obtained from Santa Cruz Biotechnology (Texas, USA). Anti-Prosurfactant Protein C (proSP-C) Antibody was obtained from MilliporeSigma (St. Louis, USA).

Cell culture

Mouse macrophage cell line Raw 264.7 cells was purchased from American Type Culture Collection (ATCC, Manassas, VA) and maintained in Dulbecco's Modified Eagle Medium (DMEM) with 10% fetal bovine serum (FBS), 1% penicillin, and streptomycin (100 μg/mL). Mouse lung epithelial cell line MLE-12 cells was obtained from ATCC (Manassas, VA) and maintained in DMEM/F-12 medium with 2% FBS, 1% penicillin, and streptomycin (100 μg/mL). All cells were cultured at 37 °C in a 5% CO_2 atmosphere.

Primary alveolar macrophages (AMs) were isolated from the lung bronchoalveolar lavage fluid (BALF) of C57BL/6 mice as previously described[1]. Briefly, after tracheostomy, mouse lungs were lavaged three times with 0.8 mL of PBS containing 2% FBS and 10 mM ethylenediaminetetraacetic acid (EDTA). The retained BALF was centrifugated at 300 g for 10 min to obtain BAL cells. Subsequently, cells were treated with ACK lysing buffer for 5 min, washed three times with PBS and cultured in DMEM supplement with 10% FBS.

Primary neutrophils were isolated from the bone marrow of C57BL/6 mice according to classical Percoll density gradient centrifugation method[2] and cultured in the RPMI-1640 medium supplement with 10% FBS.

Animals

Male C57BL/6 mice, 6–8 weeks old (Faculty of Health Sciences, University of Macau) were used in all experiments. All experiments were performed under approved by The Animal Ethics Committee of University of Macau (Ethical request No. UMARE-010-2021).

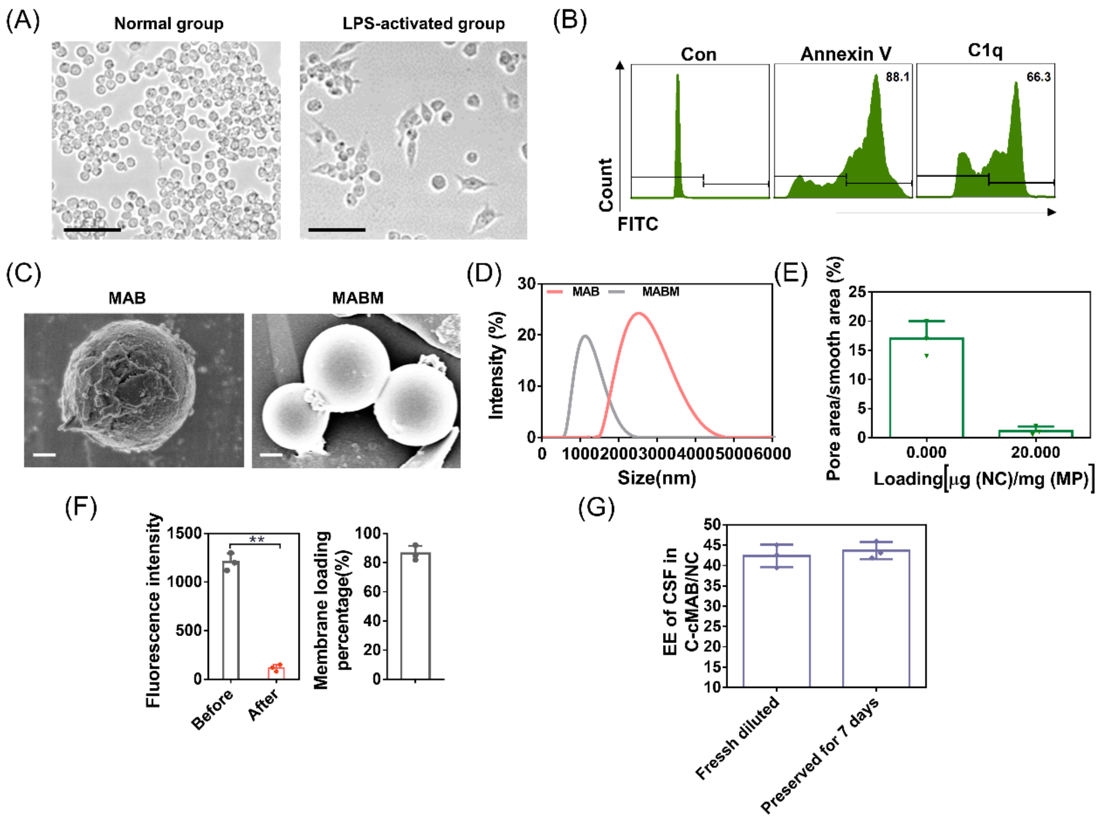

Figure S1. (A) Morphology of RAW 264.7 cells before and after LPS stimuli. scale bars, 50 μm. (B) Flow cytometry analysis of Annexin V-positive population and C1q-positive population in MABs (n=3). (C) Representative SEM images of MABs and MABMs. scale bars, 1 μm. (D) Size distributions of MABs and MABMs measured by DLS (n=3). (E) Porosity measurement of PLGA MPs and PLGA/NCs after MABM coating. Three images were used for triplicate analysis. (F) The fluorescence intensity of Cy5.5-labeled MABMs before and after coating process (n=3). (G) EE of CSF chains in C-cMAB/NCs after preserving for 7 days (n=3). $^{**}p < 0.01$.

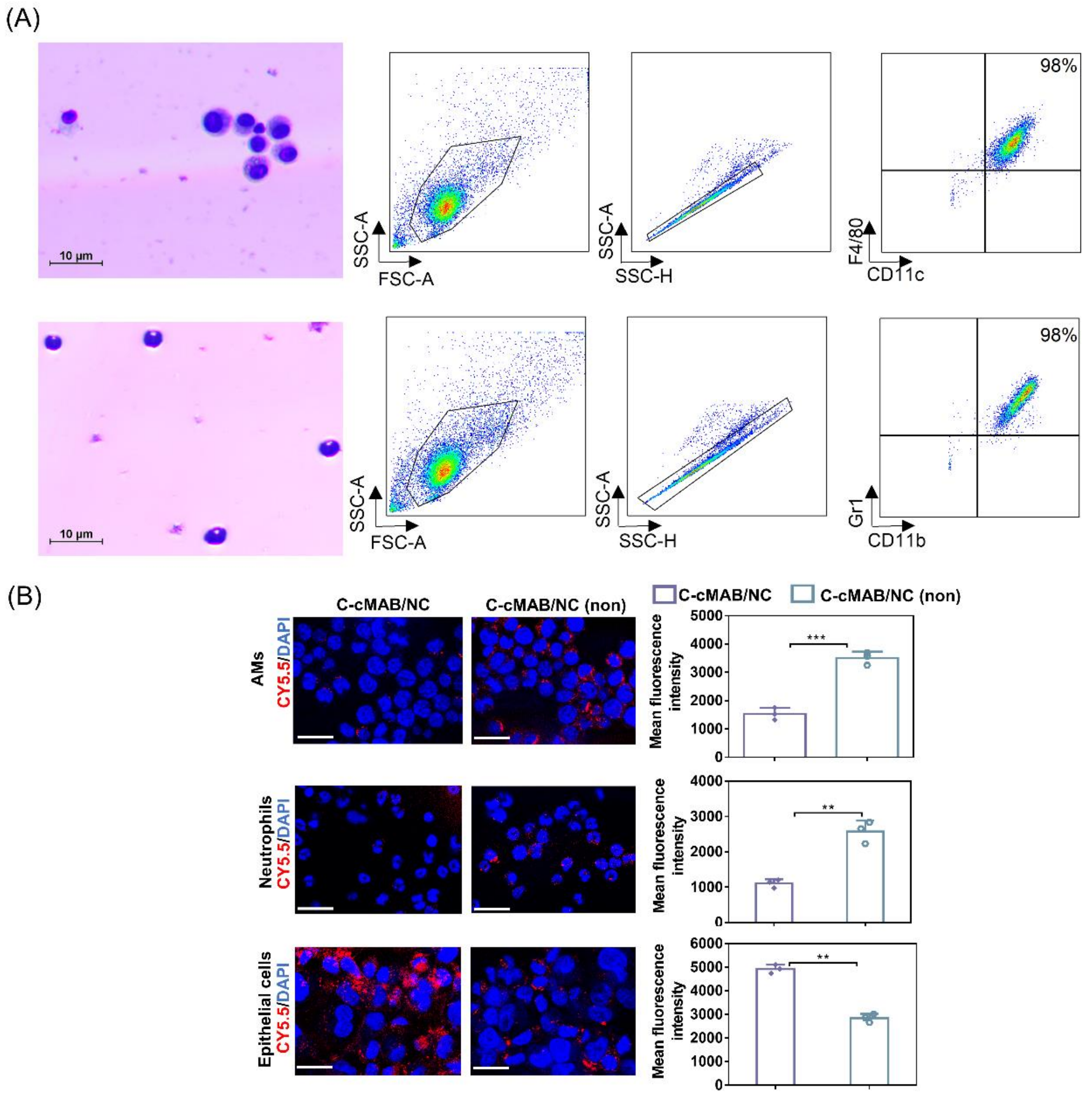

Figure S2. (A) Identification of isolated primary AMs and neutrophils by Diff-Quick staining method and flow cytometry analysis. (B) Confocal images and co-localization analysis of primary AMs, neutrophils and MLE-12 cells incubated with C-cMAB/NCs in the presence of MMP-9 or MMP-9 nonresponsive C-cMAB/NCs for 2 h (nuclei stained by Hoechst (blue), Cy5.5-labeled particles (red)). Three images were used for triplicate analysis. scale bar, 20 μm. **$p < 0.01$, ***$p < 0.001$.

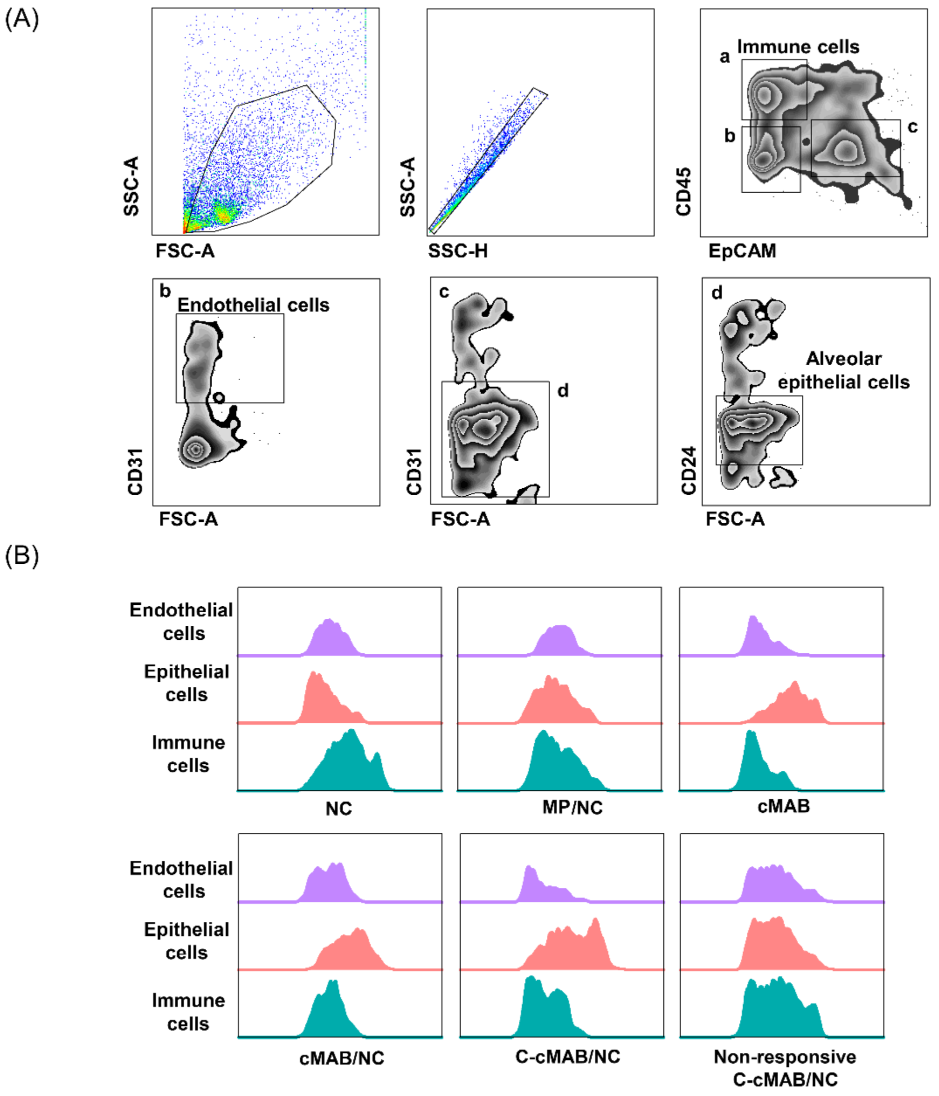

Figure S3. (A) FACS gating strategy used to identify lung immune (CD45$^+$), endothelial (CD31$^+$) and alveolar epithelial (EpCAM$^+$CD24^{lo}) cells. (B) Represented flow cytometry analysis of Cy5.5 fluorescence intensity in cells from lung tissues treated with NCs, MP/NCs, cMABs, cMAB/NCs, C-cMAB/NCs or MMP-9 nonresponsive C-cMAB/NCs.

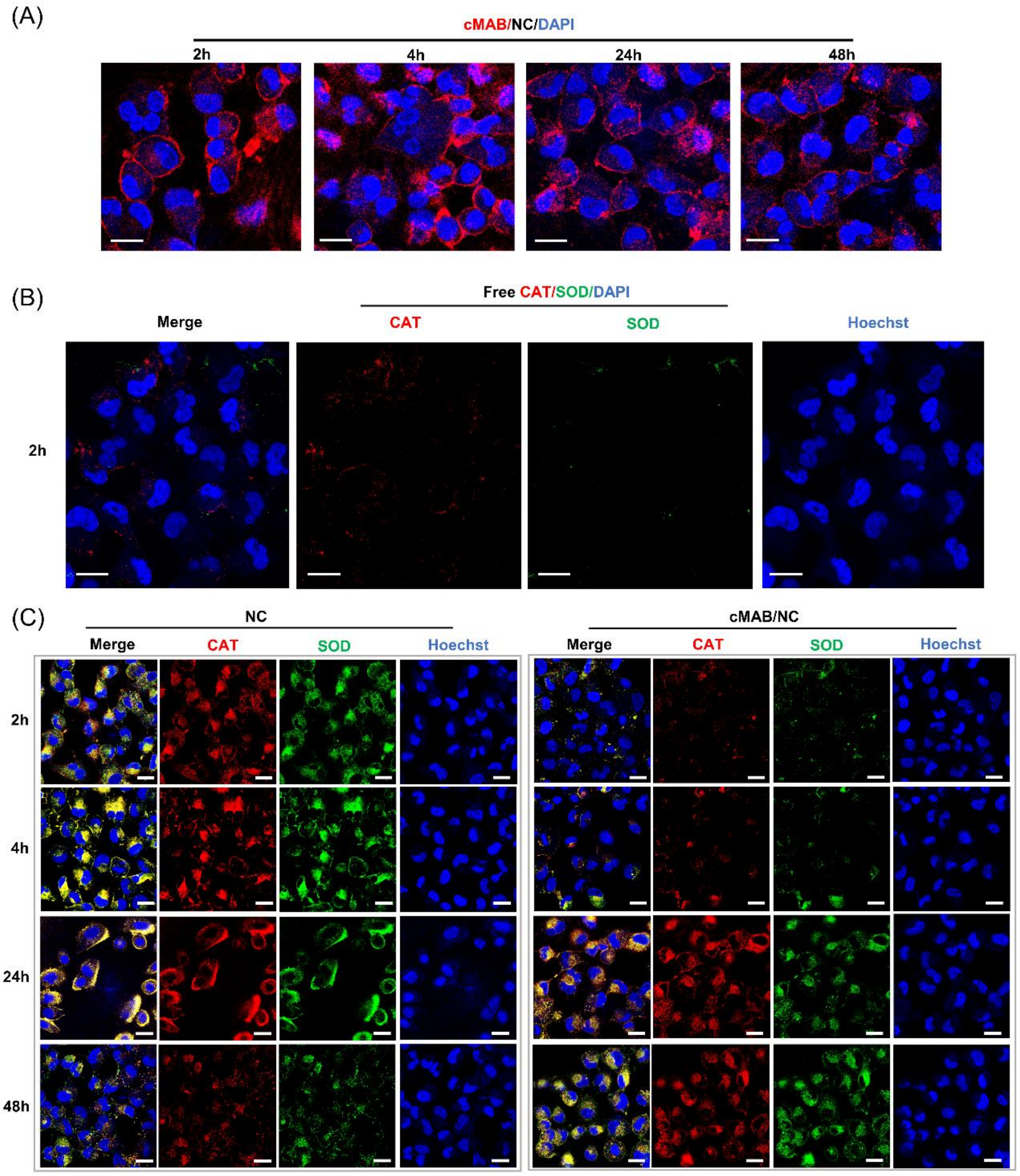

Figure S4. (A) Confocal images of Cy5.5-labeled cMABs after 2, 4, 24, 48 h of incubation (nuclei stained by Hoechst (blue), Cy5.5-labeled cMABs (red)). (B) Confocal images of MLE-12 cells taken for qualitative free Cy5.5 labeled CAT or FITC-labelled SOD after a 2-h incubation. (C) Confocal images of MLE-12 cells taken for qualitative NCs release from the cMAB/NCs after 2, 4, 24, 48 h of incubation (Nuclei stained by Hoechst (blue), Cy5.5-labeled CAT (red) and FITC-labelled SOD (green)). Sole NCs were used for comparison. scale bar, 20 μm.

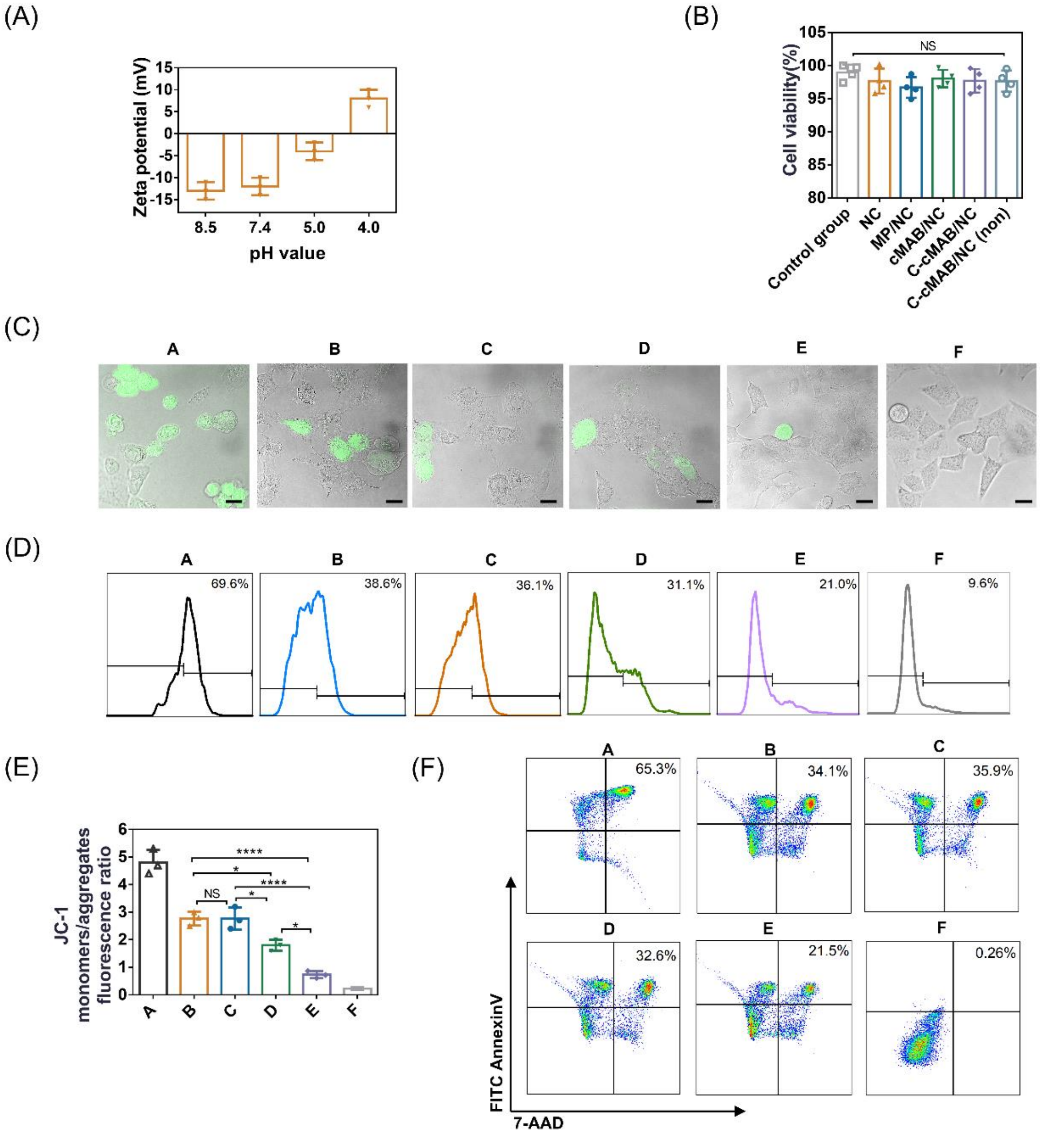

Figure S5. (A) Charge reversal of NCs at different pH values. (B-F) Cell viabilities (n=4) (B), Intracellular ROS level (C), Flow cytometry analysis for the mtROS level (D), Image-based JC-1 staining analysis (E) and Flow cytometric analysis for the apoptosis (F) of MLE-12 cells treated with indicated treatments. scale bar, 20 µm. n=3. A, H_2O_2 + PBS; B, H_2O_2 + NCs; C, H_2O_2 + MP/NCs; D, H_2O_2 + cMAB/NCs; E, H_2O_2 + C-cMAB/NCs in the presence of MMP-9; F, PBS. $*p < 0.05$, $****p < 0.0001$, n.s., not significant, $p > 0.05$.

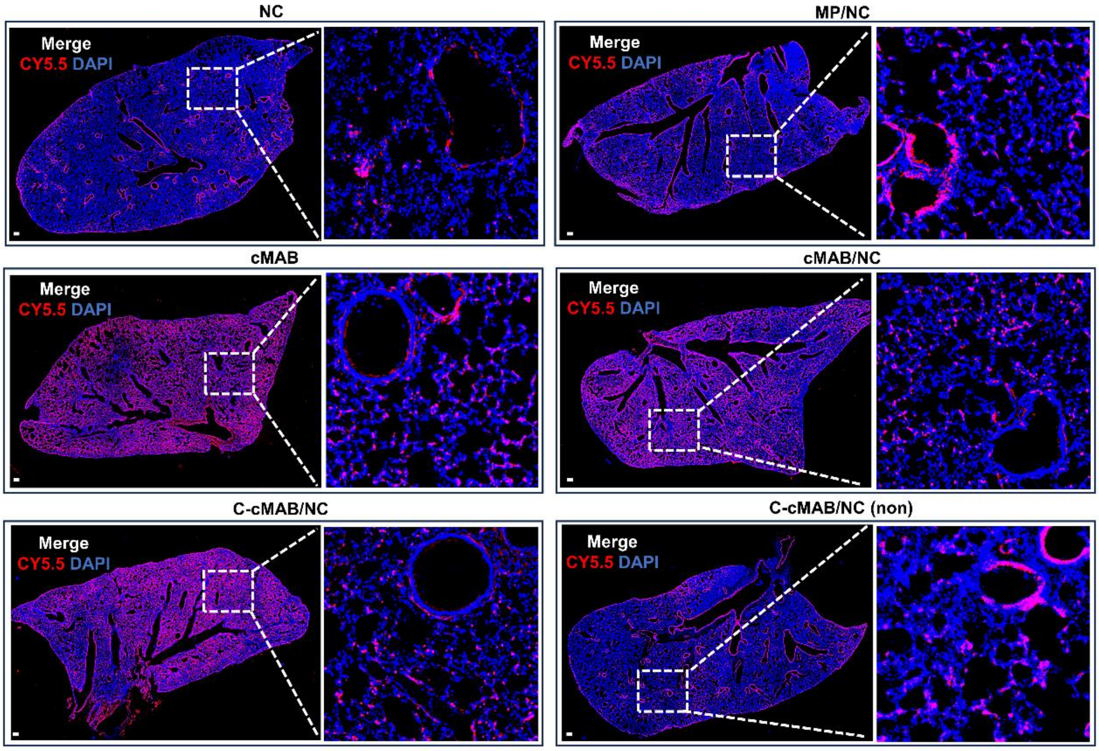

Figure S6. Localization of Cy5.5-labeled formulations in the mouse left lungs (nuclei stained by Hoechst (blue), Cy5.5-labeled formulations (red)). scale bar, 100 µm. (Inset) zoomed-up area of previous image.

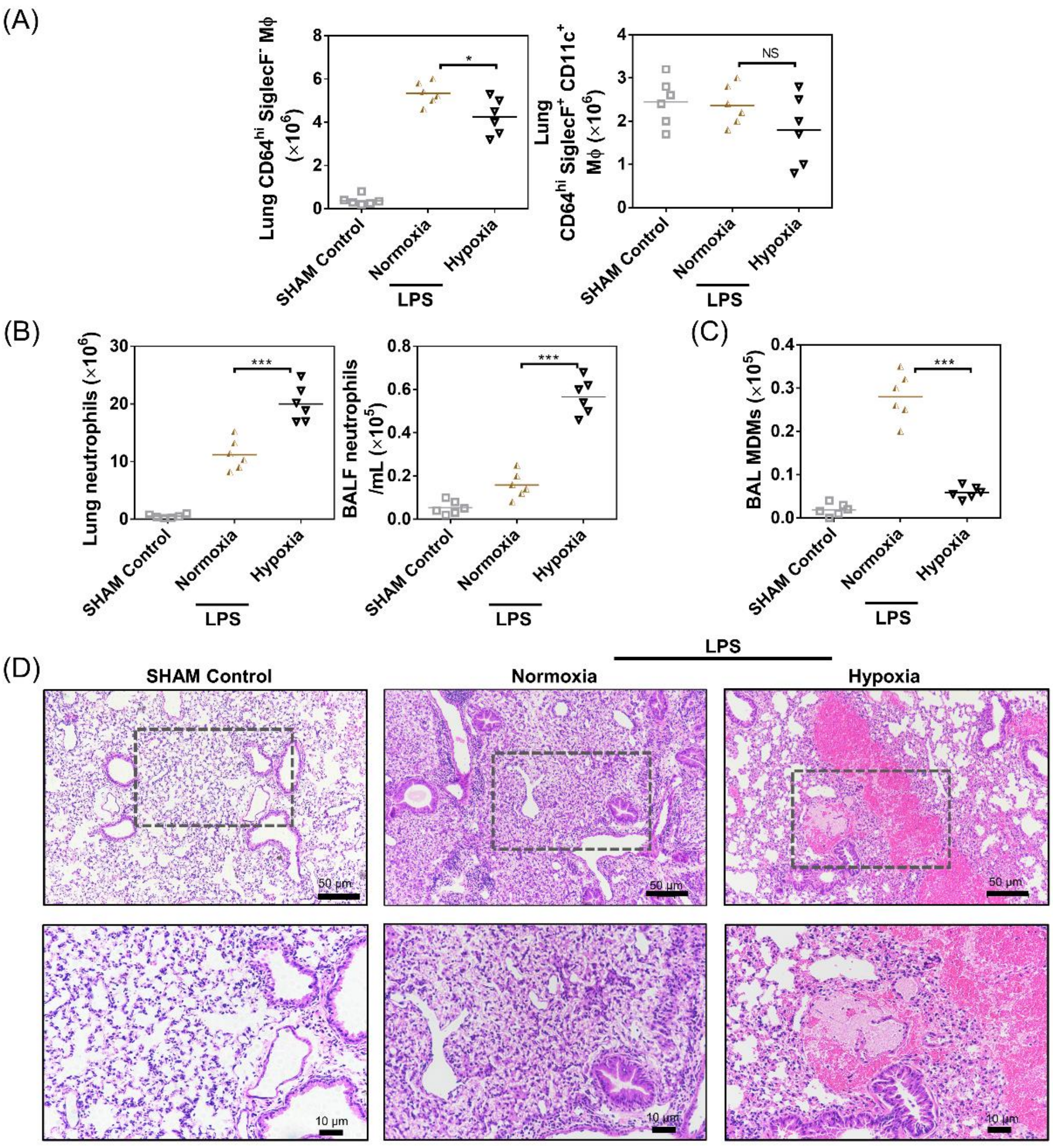

Figure S7. Establishment of LPS-treated hypoxic mouse models. (A-D) CD64^{hi}SiglecF^{-} macrophages and CD64^{hi}SiglecF^{+}CD11c^{+} macrophage numbers in the lung (A), Neutrophil numbers in the lung and BAL (B), BAL-recovered MDM numbers (C) and H&E staining images (D) from healthy mice and mice treated with LPS and housed in normoxia or hypoxia (n=6). *$p < 0.05$, ***$p < 0.001$, n.s., not significant, $p > 0.05$.

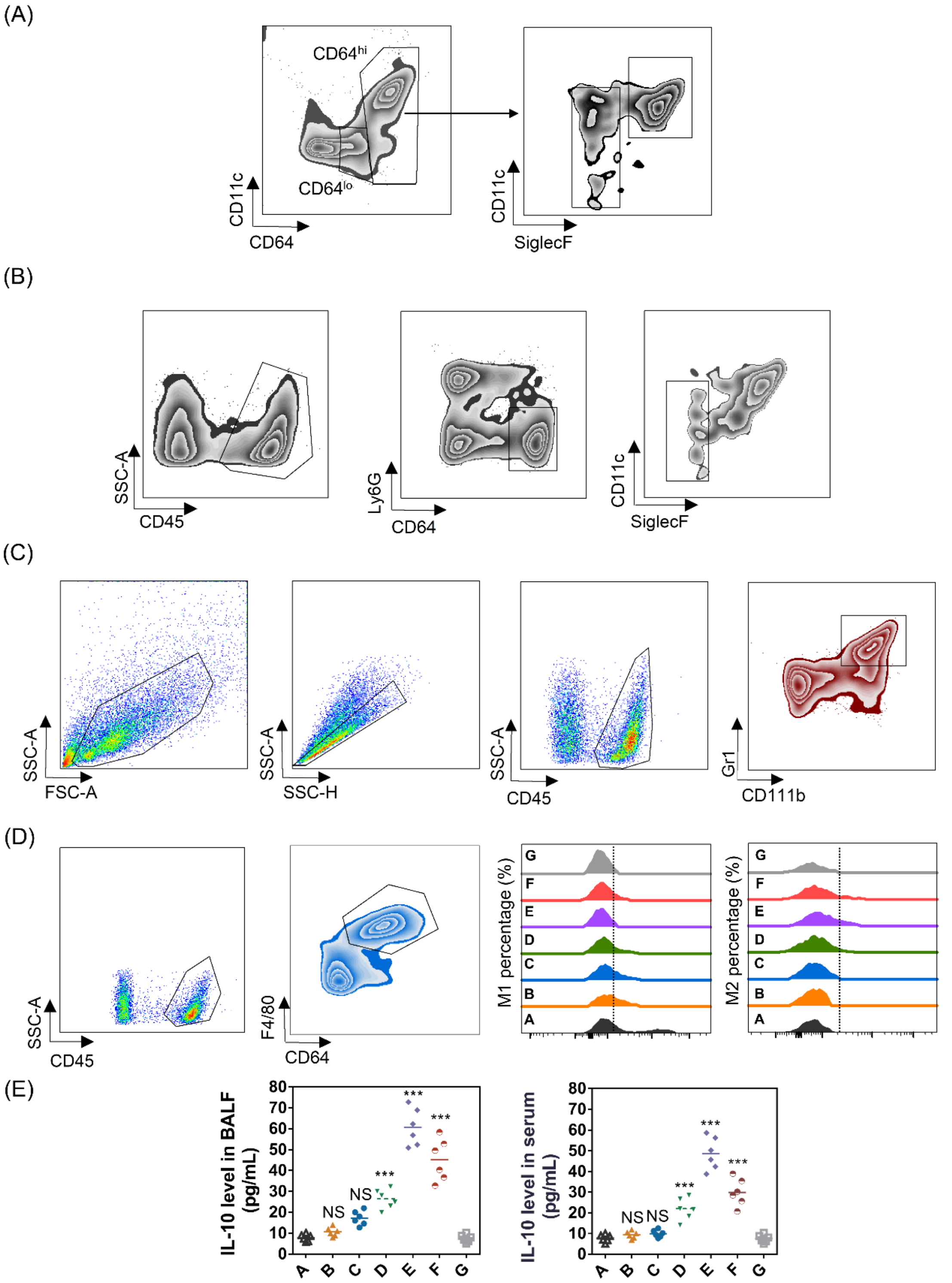

Figure S8. (A) FACS gating strategy used to identify CD64^{hi}SiglecF^{-} macrophages and CD64^{hi}SiglecF^{+}CD11c^{+} macrophages in the lung tissues. (B) FACS gating strategy used to identify CD45^{+}Ly6G^{-}CD64^{hi}SiglecF^{-} MDMs in the BAL. (C) FACS gating strategy used to identify CD45^{+}CD11b^{+}Gr1^{+} neutrophils. (D) FACS gating strategy used to identify CD64^{hi} macrophages in the lung tissues and flow cytometry histograms of the expression of CD80 (M1 macrophage biomarker) and

CD206 (M2 macrophage biomarker) on macrophages. (E) IL-10 level in the BALF and serum from LPS-challenged hypoxic mice after indicated treatments (n=6). A, hypoxic + LPS + PBS; B, hypoxic + LPS + NCs; C, hypoxic + LPS + MP/NCs; D, hypoxic + LPS + cMAB/NCs; E, hypoxic + LPS + C-cMAB/NCs; F, physical mixture of CSF and cMAB/NCs; G, sham control. ***$p < 0.001$, n.s., not significant, $p > 0.05$.

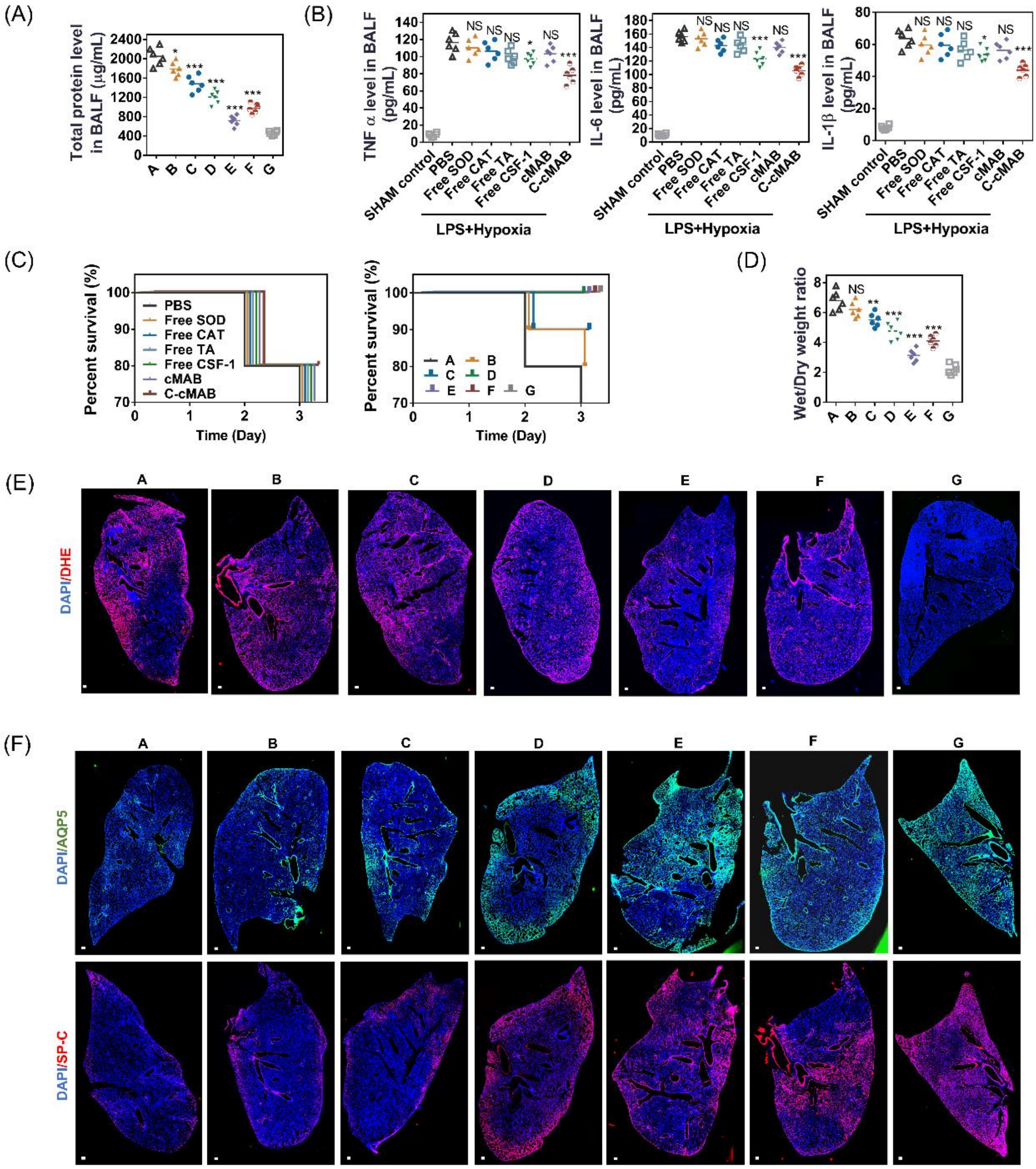

Figure S9. (A-G) Total protein level in BALF (A), Pro-inflammatory cytokines including TNF α, IL-6 and IL-1β secretion in BALF (B), Survival curve of mice with hypoxic ALI after treatment with PBS, free SOD, free CAT, free TA, free CSF-1, cMAB carrier, C-cMAB carrier (left) or NCs loaded

formulations (right). Survival analysis was performed using a log-rank test (n=10) (C), Wet/dry weight ratio (D), Representative DHE staining of lung tissues (E) and Representative IF images of AQP5 and SP-C expression in lung tissues (F) form mice treated with indicated formulations (n=6). Nuclei stained by Hoechst (blue), AQP5 (green), DHE dye or SP-C (red). scale bar, 100 μm. A, hypoxic + LPS + PBS; B, hypoxic + LPS + NCs; C, hypoxic + LPS + MP/NCs; D, hypoxic + LPS + cMAB/NCs; E, hypoxic + LPS + C-cMAB/NCs; F, hypoxic + LPS + physical mixture of CSF and cMAB/NCs; G, sham control. $*p < 0.05$, $**p < 0.01$, $***p < 0.001$, n.s., not significant, $p > 0.05$.

Table S1. Physicochemical properties of micro-formulations (n=3).

Form.	$D_{v,50}$ (Span)	$MMAD_e$ (μm)	EE for SOD (%)	EE for CAT (%)
MP	12.4±0.2 (2.1±0.008)	3.5±0.1	N/A	N/A
MP/NC	13.3±0.3 (2.6±0.006)	3.7±0.2	75.2±3.1	81.0±4.2
cMAB/NC	14.7±0.2 (2.2±0.007)	3.8±0.2	80.6±4.1	85.2±3.2
C-cMAB/NC	16.0±0.2 (2.3±0.006)	3.9±0.3	79.3±4.6	84.9±4.2

List of abbreviations

Abbreviation	Definition
ACK	Ammonium-Chloride-Potassium
AECs	Alveolar epithelial cells
AM	Alveolar macrophage
ARDS	Acute respiratory distress syndrome
BAL	Bronchoalveolar lavage
BALF	Bronchoalveolar lavage fluid
BCA	Bicinchoninic acid
CAT	Catalase

CCK-8	Cell counting kit-8
CLSM	Confocal microscope
cMAB	Chimeric macrophage apoptotic body membrane
CSF	Macrophage growth factor colony-stimulating factor
Cy5.5	Sulfo-Cyanine5.5
DBCO-NHS	Dibenzocyclooctyne-N-hydroxysuccinimidyl ester
DLC	Drug loading capacity
DLS	Dynamic light scattering
EE	Encapsulation efficiency
FBS	Fetal bovine serum
FI	Fluorescence intensity
FITC	Fluorescein isothiocyanate
HALI	Hypoxic acute lung injury
H&E	Hematoxylin and eosin staining
HPMC	Hydroxypropyl methylcellulose
IF	Immunofluorescence
IVIS	In vivo imaging system
LPS	Lipopolysaccharide
MAB	Macrophage apoptotic body
MABM	Macrophage apoptotic body membrane
MDM	Monocyte derived macrophage
MLE-12	Mouse lung epithelial cells
$MMAD_e$	Experimental mass median aerodynamic diameter
MMP-9	Matrix metalloproteinase 9
MP	Microparticle
mtROS	Mitochondrial reactive oxygen species
NCs	Nanocomplexes
NGI	Next generation impactor
NLRP3	NOD-like receptor thermal protein domain associated protein 3
PLGA	Poly(lactic-co-glycolic) acid
PVA	Polyvinyl alcohol
PVP-K12	Polyvinyl pyrrolidone K-12
ROS	Reactive oxygen species
RPMI	Roswell park memorial institute
SEM	Scanning electron microscope
SOD	Superoxide dismutase
TA	Tannic acid
TEM	Transmission electron microscope

Supplemental references

1. Liu, C.; Xi, L.; Liu, Y.; Mak, J. C. W.; Mao, S.; Wang, Z.; Zheng, Y. An Inhalable Hybrid Biomimetic Nanoplatform for Sequential Drug Release and Remodeling Lung Immune Homeostasis in Acute Lung Injury Treatment. *ACS Nano.* **2023**, *17*, 11626-11644.
2. Myerson, J. W.; Patel, P. N.; Rubey, K. M.; Zamora, M. E.; Zaleski, M. H.; Habibi, N.; Walsh, L. R.; Lee, Y.-W.; Luther, D. C.; Ferguson, L. T. Supramolecular arrangement of protein in nanoparticle structures predicts nanoparticle tropism for neutrophils in acute lung inflammation. *Nat. Nanotechnol.* **2022**, *17*, 86-97.
